# Understanding the influencing factors of telemedicine service adoption in post-pandemic Bangladesh: An extended TAM and UTAUT2 model analysis

**DOI:** 10.1371/journal.pone.0361691

**Published:** 2026-10-28

**Authors:** Mohammad Rakibul Islam Bhuiyan, Md Abu Sayem, Niaz Makhdum

**Affiliations:** 1 Department of Management Information Systems, Begum Rokeya University, Rangpur, Rangpur, Bangladesh; 2 Department of Local Government and Urban Development, Jatiya Kabi Kazi Nazrul Islam University, Trishal, Mymensingh, Bangladesh; 3 Department of Public Administration, Begum Rokeya University, Rangpur, Rangpur, Bangladesh; University of Southampton, MALAYSIA

## Abstract

In the post-pandemic period, telemedicine services are a new addition to health sectors that facilitate bridging the gap for patients in rural and marginalized areas. The study aims to comprehend the different influential factors associated with adopting telemedicine services in Bangladesh. The study developed several hypotheses from two technology acceptance models, including the Unified Theory of Acceptance and Use of Technology 2 (UTAUT2) and the Technology Acceptance Model (TAM). An explanatory research design was followed using a quantitative approach. The study developed a semi-structured questionnaire to collect primary data. It used Partial Least Squares-Structure Equation Modeling (PLS-SEM) to identify influential factors in adopting telemedicine services in Bangladesh. Key findings indicate that trust (β = 0.469, p < 0.001) and perceived usefulness (β = 0.418, p < 0.001) are the strongest predictors of behavioral intention to use telemedicine. Perceived ease of use (β = 0.325, p < 0.05) also significantly was associated with resistance to use, emphasizing the importance of user-friendly interfaces. However, resistance to use didn’t find any significant relationship with behavioral intention (β = 0.046, p > 0.05), suggesting that the urgency created by the COVID-19 pandemic may have overridden previous resistance to telemedicine. The study also highlights the negative influence of perceived risk (β = 0.286, p < 0.05) and technology anxiety (β = 0.242, p < 0.05) on resistance to use, pointing to concerns about data security and digital literacy. Surprisingly, social influence (p > 0.05) and facilitating conditions (p > 0.05) were not found to have a significant impact on resistance to use. This research underscores the importance of addressing security concerns, improving digital literacy, and enhancing the user-friendliness of telemedicine platforms to promote widespread adoption from the implication perspective. These insights are critical for policymakers and healthcare providers aiming to take immediate policy-level initiatives expanding telemedicine services in Bangladesh.

## Introduction

In 2024, the global internet users have reached around 5.35 billion (66% of the world population) and are expected to reach 7.9 billion by 2029 [1]. Bangladesh has witnessed this global trend, with 77.36 million internet users in 2024, a 1% increase from the previous year, and a penetration rate of 44.5% of the total population [2]. This technological advancement has accelerated the simplification of service processes in developing countries, leading to the development of various aspects of human life, including healthcare. This has enabled citizens to receive quality health services at their doorsteps. The COVID-19 pandemic has leveraged advanced technologies to initiate a ‘contactless society’ throughout the world reducing the health risks resulting the term ‘telemedicine’ has spread rapidly among the people [3]. Telemedicine systems have successfully tackled the COVID-19 pandemic in both developed and developing countries, including Bangladesh, and due to its easy accessibility, people are highly interested in using this system to receive services in the post-pandemic era.

Bangladesh is the most densely populated country, and around 68.34% of people live in rural areas [4]. The Bangladesh hospital facilities are inadequate where hospital bed and patient ratio 1:990 and ratio have exacerbated in rural areas. The health care infrastructures are highly poor and ill-equipped in rural areas, resulting in most of the doctors being city-based, and they mostly shifted to urban or city around 1 or 2 years. Consequently, the telemedicine health system has become popular among the people of Bangladesh because it facilitated the patients to receive quality health services from their comfort places. Telemedicine enhances healthcare accessibility by enabling families to overcome mobility challenges, minimize time spent away from work, avoid waiting times, and reduce the risk of exposure to contagious infections that are commonly encountered in clinics and contribute to lower patient satisfaction [5]. The study stated the present scenario of rural health care center that found several challenges including shortage of doctors, the substantial distance between health center and patients house, unequal doctor and patient’s ratio gap, and fragmented health care delivery [6].

Additionally, the advancement of digital technologies has facilitated the process-based health administrative system for becoming more effective and reaching the marginalized communities [7] and developing the regional structural deficiencies with reducing geographical distances [6]. However, telemedicine allows healthcare professionals to improve and expand the delivery of care beyond traditional healthcare facilities, resulting in cost reduction and the creation of new benefits for physicians and patients [5]. Telephone and video-based communication methods have gained significant attention, as seen by the impact of the COVID-19-related healthcare issues. For example, several countries including India launched telemedicine service with ‘National Telemedicine Network’, China implemented telemedicine with Golden Health Network, and International MedioNet of China, Cuba introduced telehealth with including Virtual University of Health Care, and Virtual Library of Health Care to provide essential health services for people living rural or marginalized areas [7]. The current pandemic has significantly strained the healthcare system and has highlighted the crucial role of telemedicine in maintaining an effective healthcare system [8]. Telemedicine services cover the several functions including diagnosis, therapy, patient monitoring, and consultations for making the medical decision [9]. However, the study aims to explore the influential factors associated with the adaptation of Telemedicine Services (TLS) in Bangladesh’s post-pandemic era, using both the Technology Acceptance Model (TAM) and Unified Theory of Acceptance and Use of Technology 2 (UTAUT2). A number of studies have examined the behavioral intention of Telemedicine Services (TLS) applying these models [3,10–13].

## Conceptual framework

Several models and theoretical underpins have been developed for exploring the different factors of technology acceptance among the users in last century to adopt the people in advanced service delivery tools [14]. The effectiveness of these models for various information technology-based applications has been verified multiple times [15]. The TAM developed by Davis is extensively regarded as the most well-established framework for understanding technology acceptance [16]. The TAM has its roots in the disciplines of sociology and psychology, has emerged as the predominant model employed in numerous research investigations [17]. The TAM is an extensively utilized framework in the field of technology adoption with perceived usefulness and perceived ease of use and these constructs have been extensively studied and applied in various technological contexts [16].

The UTAUT framework is another technology acceptance model developed with various constructs by Venkatesh and others in 2003 to harness the technology in service delivery process [18]. The study found several influential factors of behavioural intention toward mobile health (mHealth) services among the elderly population in Bangladesh [19]. The UTAUT model was expanded by Hoque and Sorwar to include two additional variables, namely technology anxiety and resistance to change [19]. This extension aimed to enhance the understanding of users’ behavioral intention to adopt mHealth, and a similar extended UTAUT model was applied by Shiferaw et al. to healthcare providers’ acceptance of telemedicine in a low-resource setting [20]. Previous research has conducted multiple studies to investigate the UTAUT2 as the theoretic framework for understanding technology acceptance and usage [12]. Baudier et al. investigated a research where the decision to exclude the price value and hedonic motivation variables from their research [10], as they were deemed unsuitable for the purposes of the study [11]. Though much research has been conducted on technology acceptance utilizing the UTAUT2 model [21], there is a scarcity of research investigating customer satisfaction utilizing this model.

Ammenwerth suggested that two such models that have garnered attention are the TAM and the UTAUT2 model [22]. While both models offer valuable insights into understanding user behavior, they focus on different aspects of technology acceptance [23]. The integration of TAM and UTAUT2, as applied to telemedicine adoption during the COVID-19 pandemic in an emerging market, provides a comprehensive framework for analyzing telemedicine adoption in the post-pandemic context of Bangladesh [24]. While TAM focuses on the intrinsic beliefs of users (e.g., usefulness and ease of use), UTAUT2 and its extensions emphasize external factors such as Social Influence, Facilitating Conditions, and Trust, which are critical for understanding the context-specific challenges in telemedicine adoption [25]. In the case of Bangladesh, these models complement each other by addressing both internal and external factors that influence adoption [19,26].

The combined use of TAM and UTAUT2 is particularly justified in the study of telemedicine adoption in post-pandemic Bangladesh due to the complexity and multi-dimensional nature of adoption behaviors [27]. This integration allows for a comprehensive analysis of not only user acceptance but also the external factors that could facilitate or hinder telemedicine adoption in Bangladesh [6]. The extended model also considers the unique challenges posed by the country’s healthcare infrastructure, socio-cultural context, and technological readiness [28]. These factors—such as social influence, trust, and facilitating conditions—are particularly important in a post-pandemic era, where the rapid growth of digital health services necessitates a more holistic approach to understanding user behavior [29].

To meet the research objectives, the TAM model was employed, utilizing key components consisting of Perceived Usefulness (PU), Perceived Ease of Use (PEOU), Technology Anxiety (TA), Resistance to Use (RU), and Behavioral Intention to Use (IU), as illustrated in Fig 1 for the development of this research model. Additionally, several dimensions from UTAUT 2, including Facilitating Conditions (FC), Social Influence (SI), Perceived Risk (PR), and Trust, were incorporated into the conceptual study model depicted in Fig 1.

**Fig 1:**
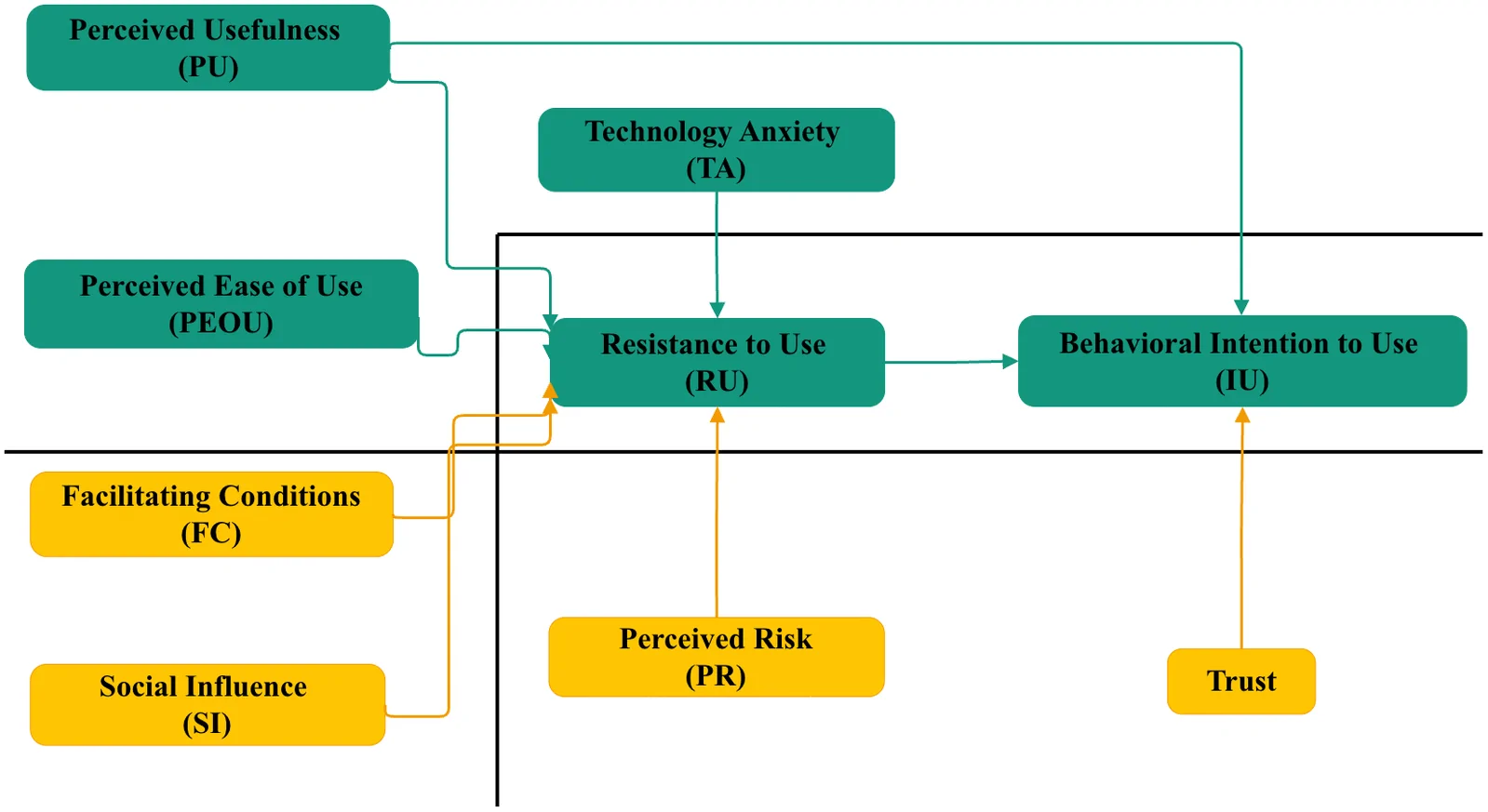
Research model-combined TAM and UTAUT 2 constructs (author’s contribution).

### Hypothesis development

#### Perceived usefulness (PU).

Based on the TAM proposed by Davis in 1989, the adoption and usage of information technology are affected by the person’s behavioral usage it [16]. The value of a particular service or product, is known to be influenced by various factors [30]. The TAM has garnered significant attention and utilization in real-world settings, as well as further exploration and examination in academic circles, particularly within the realm of information management, over the past decade [22]. The attitudes of physicians towards the usage of telemedicine technology has been examined by Shadangi et al. [31]. They utilized the TAM as a framework for their investigation and expanded upon this model by incorporating additional factors such as technology infrastructure support and risk. The impact of risk and technology infrastructure on a doctor’s attitude towards utilizing TLS is a significant area of study [26]. A TAM study discovered that PU plays a crucial role in users’ intention to adopt telemedicine [32]. However, it was found that perceived risk, including financial and privacy risk, has a detrimental impact on the intention to adopt e-services [33]. Hypothesis is stated that:

*H1: PU has an association with Resistance to Use (RU).*

#### Perceived ease of use (PEOU).

The construct of PEOU plays a noteworthy role in shaping user satisfaction in the context of mobile health (m-Health) [34] and mobile commerce (m-commerce) [35]. PU is a key factor in the TAM and is frequently linked to the concept of PEOU. PEOU is a robust predictor of technology acceptance [23]. The researchers employed the TAM to examine the relationship between PU and attitudes towards telehealth. Within the scope of this investigation, the concept of positive attitude encompasses both the state of being satisfied and possessing a high level of favorability. Given the information provided in the description, hypotheses are stated that:

*H2: PEOU has a positive relationship with RU.*

#### Technology anxiety (TA).

The current study incorporates the TAM as its theoretic framework, along with the inclusion of supplementary variables such as resistance to change, technological anxiety, as well as privacy concerns. Given the rapid progress in the field of science and technology, it is imperative to investigate and comprehend the inclination of individuals to embrace novel technologies [36]. TA refers to the emotional state characterized by fear or apprehension that individuals may encounter when they contemplate or initiate the use of a technology oriented that is not familiar to them [37]. It was suggested that individuals may experience feelings of anxiety when considering the use of TLS [19]. This apprehension could potentially hinder their willingness to engage with such services. Therefore, hypotheses are stated that:

*H3: Technology Anxiety (TA) has influenced to Resistance to Use (RU).*

#### Perceived usefulness (PU) directly influences behavioral intention to use (IU).

The concept of PU is defined as the person’s belief in the potential of a system to improve one’s performance [16]. Within the scope of this research, it was necessary to redefine PU due to the evolving interpretation of the term in relation to the usefulness of a system for patients. This redefinition was prompted by the recognition that the previously established definitions of PU may no longer fully capture its current significance [38]. Patients perceive the potential benefits of embracing TLS to be contingent upon the achievement of several key factors. These factors include expedited delivery of healthcare services, cost-effectiveness in terms of medical examinations, enhanced documentation practices, and reduced service time for healthcare provision. Thus, the hypothesis was formulated as follows:

*H4: Perceived Usefulness (PU) directly influences Behavioral Intention to Use (IU).*

#### Facilitating condition (FC).

It refers to the presence of technical and organizational infrastructure that is essential for enabling and supporting the usage of a system. The study found the significant association between facilitating condition and its impacts on the user’s intention to use technology [39]. In addition, the users who use smartphones have found positive relationship among the usage and adoption of health information systems [40] and mobile health services [19] with FC. FCs play a crucial role in comprehending users’ general awareness of mHealth services [34] and the acceptance behavior of TLS among elderly individuals [40]. Thus, the formation of the hypothesis can be described as follows:

*H5: Facilitating Condition (FC) influences the Resistance to Use (RU).*

#### Social influence (SI).

Social Influence refers to how users’ perceptions, thoughts, beliefs, and behaviors are influenced to accept new technology in the service delivery process [41]. In present time, individuals frequently reside within joint family structures, wherein they rely on one another for various social and economic needs in the developing nations. The utilization of TLS within this particular context was not only be observable by individuals within the user’s social network, but their perspectives and endorsements might also serve as a motivating factor for the end-users to engage with TLS [42]. While certain studies on m-health, have found that SI does not have an important influence on user satisfaction [34]. The hypothesis stated that:

*H6: There is a favorable influence between Social Influence (SI) to Resistance to Use (RU).*

#### Perceived risk (PR).

It is a construct that encompasses two fundamental elements: the uncertainty experienced by individuals and their perception of the severity of potential negative outcomes resulting from a particular action [33]. Within the realm of healthcare, the presence of a specific vulnerability that patients may face. This vulnerability stems from the potential risks that are associated with their personal health [43]. Individuals exhibit a tendency to modify their behavior in order to mitigate risks [44]. All necessary individual health information are shared with the physician during a telemedicine in order to receive appropriate treatment [45]. The findings of a research indicated that there was an adverse association between perceived privacy risk and the intention to use TLS [44]. In other words, when individuals perceive a higher level of privacy risk, they are less likely to have the intention to use telemedicine. It has been observed that individuals who express higher levels of apprehension regarding the potential misuse of their personal data are less inclined to utilize TLS [43]. These findings highlight the importance of privacy and data security in telemedicine research. Thus, the following hypothesis emerges:

*H7: Perceived Risk (PR) has a negative association to Resistance to Use (RU).*

#### Resistance to use (RU) with behavioral intention to use (IU).

As per the TAM proposed by Davis in 1989, the adoption and utilization of information technology are influenced by the behavioral intention of individuals to use it. Previous research has also corroborated the notion that individuals’ resistance towards technology has a negative impression on their intention to adopt and utilize a novel technological innovation [19,46]. Past research has indicated that there is a positive association among the person’s willingness to use a particular technology and their likelihood of engaging in the associated behavior [16,18]. The phenomenon of this effect has been observed and documented in the field of TLS as well. According to the extended research on technology acceptance, specifically the intention-behavior literature [16,18]. Thus, based on our observations and existing knowledge, researchers suggest the following hypothesis:

*H8: Resistance to use (RU) interconnects to IU.*

#### Trust with behavioral intention to use.

eHealth services have been widely recognized to be influenced by the level of trust for the assessment acceptance [43]. In the realm of TLS, the concept of trust has been subject to multiple interpretations. The health-care system, which includes hospitals, can be classified as an institution. Institutional trust refers to the ability of patients to place trust in healthcare providers even in the absence of personal knowledge about the individual health workers [47]. There is a suggested association between patient trust in TLS providers and the clinical or technical competence which includes factors such as listening and respect [48]. Within the realm of research pertaining to the examination of the telemedicine acceptance services with trust when it comes to participating in actions such as medical prescription or the procurement of medicine [43,49]. Thus, the hypothesis is formulated as follows:

*H9: There is a positive impression between Trust and Behavioral Intention to Use (IU).*

## Materials and methods

### Study design

The explanatory research design was followed utilizing a quantitative research approach to conduct the study. The basic framework for this present study is the TAM and the UTAUT2 theories, which are widely adopted in the studies of technology acceptance and user behavior in health care [15,50–52].

### Sampling technique

The people aged 18 and above in Bangladesh who took health care services through telemedicine in post-pandemic period is the targeted population of this study. As a developing country, where most of the people lives in rural areas, and have limited access to internet specially for elder people making the population of this study as “hard-to-reach” populations [53–55]. Common methods for sampling hard-to-reach populations include convenience sampling and snowball sampling [55–57]. Consequently, researchers employed a convenience sampling method to identify initial respondents and utilized snowball sampling, enabling these initial respondents to distribute the questionnaire to their acquaintances who could potentially participate in the survey. The online questionnaire was distributed on Facebook, WhatsApp, and emails and a total 276 respondents attended the full survey. Numerous studies have been undertaken with nearly the same number of respondents to investigate various factors and health-related issues [58–63]. All respondents had been provided with detailed information on the study in advance and asked for informed consent.

### Data collection procedures

Data collection instrument tool was developed by a structured survey questionnaire and collected data through online platforms like social media. The questionnaire was adapted from validated constructs of the TAM and UTAUT2 models, and a few more questions were incorporated based on the recent utility of telemedicine in Bangladesh [16,23,34–36]. This survey was conducted in English and Bengali for the convenience of the respondents. The data collection period was extended by two months, starting from February to March 2024, allowing enough time to meet the set sample size.

### Survey instrument

The questionnaire comprised a sequence of sections covering various aspects related to the topic under this study. Information on demographic characteristics (age, gender, education level, income in the rural and urban areas, and easy access to the internet) was collected in the first section of the questionnaire. The following section studied various factors of the technology acceptance (PU, PEOU, TA, and RU) along with behavioral intention to use telemedicine services using a 5-point Likert scale (1 = strongly disagree; 5 = strongly agree), which was adapted from several validated scales in previous studies related to technology acceptance. A subset of constructs from the UTAUT2 model, including FC, SI, PR, and trust, were also administered on Likert scale adapted to telemedicine. Using a sample of participants, the questionnaire was pilot tested to assess clarity and reliability. Adjustments to question wording and the survey structure were made based on pilot test feedback.

### Data analysis plan

The collected data was analyzed using statistical software (Stata and Smart PLS). The study assessed both descriptive and inferential statistical tests. Descriptive statistics was used to summarize the demographic profile of the respondents. The study was assessed the different reliability and validity tests through convergent validity and discriminant validity to fit model for this study. PLS-SEM was used to measure the complex relationships among each construct of the model. This test facilitated the researcher to identify the most weighted factor to influence the dependent variable. To test the hypothesis relationships among the constructs, the structural model has been evaluated and path coefficients and p-values were further assessed considering a 0.05 level of significance to measure the strength of relationships between dependent and independent variable individually.

The R-squared (R^2^) was employed to evaluate the explanatory ability of the independent variables on the dependent variable, which describes how much variance of behavioral intention to use telemedicine could be explained. To determine the extent to which the independent variables could explain the variation in the dependent variable—the behavioral intention to use telemedicine—the R-squared (R^2^) test was used. Bootstrapping procedures with 5,000 resamples were used to determine the path coefficients’ statistical significance.

### Ethical considerations

Ethical approval (PADBRUR-RAC-001/24) for this study was obtained from the research approval committee of Department of Public Administration, Begum Rokeya University, Rangpur. Strict adherence to ethical standards was maintained throughout the course of the study. The study objectives, purpose, and ethical issues were mentioned on the first page of the questionnaire, and respondents could only fill out the questions after giving written consent to attend this study. All responses were anonymized, and data was stored securely following data protection regulations. The data was only used for this study and was only available to the research team.

## Results

The demographic information was analyzed though SPSS and construct data was analyzed through SmartPLS software for addressing the research gaps.

Table 1 presents the demographic profile of the study participants, revealing that 58% of them are male and 42% are female. The study collected the same proportion of data from urban and rural areas to get comprehensive insight into this problem. Furthermore, most of the respondents (82%) fall within the age range of 20–30, and the majority (82%) hold higher education levels. Moreover, the study also shows that around 88% of respondents have internet access to lead their daily lives in Table 1.

**Table 1:** Demographic information of the respondents.

Variables		Percentage
Gender	Male	58.0
	Female	42.0
Area of Residents	Rural	52.5
	Urban	47.5
Age of Respondents	< 20 Years	5.4
	20 to 30 Years	82.2
	30 to 40 Years	10.9
	40 to 50 Years	1.1
	> 50 Years	0.4
Educational Status	Non-literate	0.7
	Primary Education	1.1
	Secondary Education	16.3
	Higher Education	81.9
Monthly Income (BDT)	< 10000	72.5
	10000–20000	18.5
	20000–30000	4.0
	30000–40000	1.8
	>50000	3.3
Easy Access to Internet	No	12.0
	Yes	88.0

Table 2 shows the measurements of each construct to fit the model and assessed two types of validity test for the measurement of the model. Convergent validity was checked using factor loading, Cronbach’s alpha (α), the composite reliability test, and Average Variance Extracted (AVE), while discriminant validity was assessed using the Heterotrait-Monotrait ratio (HTMT). Factor loading was checked for each item to ensure it was positive and greater than 0.7 indicates the strength relationships between variables [64]. All of the standardized factor loadings meet the above-recommended threshold. In addition, the Cronbach’s alpha value of each construct have found ranged from 0.869 to 0.944 which are greater than 0.7 [65]. Furthermore, both composite reliability and AVE value range from 0.871 to 0.944 and 0.690 to 0.850 in Table 2, respectively, which all meet the recommended thresholds (>0.7 for composite reliability and >0.5 for AVE) [66].

**Table 2:** Evaluation of measurement model test result.

Variables	Items	Factor Loading	Cronbach’s Alpha	Composite Reliability	AVE
Perceived Usefulness (PU)	PU 1	0.924	0.939	0.940	0.838
	PU 2	0.920			
	PU 3	0.902			
Perceived Ease of Use (PEOU)	PEOU 1	0.878	0.916	0.918	0.786
	PEOU 2	0.921			
	PEOU 3	0.859			
Technology Anxiety (TA)	TA 1	0.791	0.872	0.873	0.693
	TA 2	0.831			
	TA 3	0.873			
Resistance to Use (RU)	RU 1	0.843	0.869	0.871	0.690
	RU 2	0.859			
	RU 3	0.788			
Facilitating Conditions (FC)	FC 1	0.918	0.922	0.924	0.798
	FC_2	0.926			
	FC 3	0.832			
Social Influence (SI)	SI 1	0.885	0.932	0.933	0.821
	SI 2	0.909			
	SI 3	0.924			
Trust	Trust 1	0.886	0.933	0.934	0.823
	Trust 2	0.926			
	Trust 3	0.909			
Perceived Risk (PR)	PR 1	0.848	0.892	0.893	0.734
	PR 2	0.840			
	PR 3	0.882			
Intention to Use (IU)	IU_1	0.915	0.944	0.944	0.850
	IU_2	0.920			
	IU_3	0.930			

Table 3 presents the HTMT test that was used to check the discriminant validity of the research model. The threshold level of this discriminant validity is less than 0.9 [67]. As shown in the Table 3, all the HTMT values of each construct were below 0.90, indicating discriminant validity in all constructs.

**Table 3:** Heterotrait-Monotrait ratio of correlation (HTMT).

	PU	PEOU	RU	Trust	TA	FC	SI	PR	IU
PU	1								
PEOU	.818	1							
RU	.627	.626	1						
Trust	.860	.797	.575	1					
TA	.434	.423	.631	.410	1				
FC	.841	.872	.604	.828	.475	1			
SI	.864	.855	.618	.811	.483	.809	1		
PR	.462	.396	.625	.408	.709	.475	.415	1	
IU	.850	.856	.577	.855	.465	.850	.892	.409	1

### Structural equation model result

PLS-SEM algorithm was employed to analyze the proposed hypothesis after ensuring all the criteria to fit the model. The result showed that the independent variables in this model can explain 78.2% of the variation in the dependent variable the behavioral intention to use telemedicine as shown in the Fig 2. A standardized path coefficient was used for analyzing the data and the result showed a P value of 0.05. The Fig 2 further demonstrates that patients’ behavioral intention to use has a substantial impact on trust and perceived usefulness (β = 0.469, P < 0.001) and (β = 0.418, P < 0.001), whereas resistance to use (β = 0.046, P > 0.05) did not show any significant relation with the dependent variable. Astoundingly, there was no direct association between the perceived usefulness and resistance to use (β = 0.017, P = 0.948). However, the study demonstrates that feelings of technological anxiety (β = 0.242, P < 0.05), perceived risk (β = 0.286, P < 0.05), and perceived ease of use (β = 0.325, P < 0.05) had a significant impact on resistance to use. As illustrated in Fig 2, facilitating conditions (β = −0.062, P > 0.05) and social influence (β = 0.146, P > 0.05) did not demonstrate any significant correlation with resistance to use.

**Fig 2:**
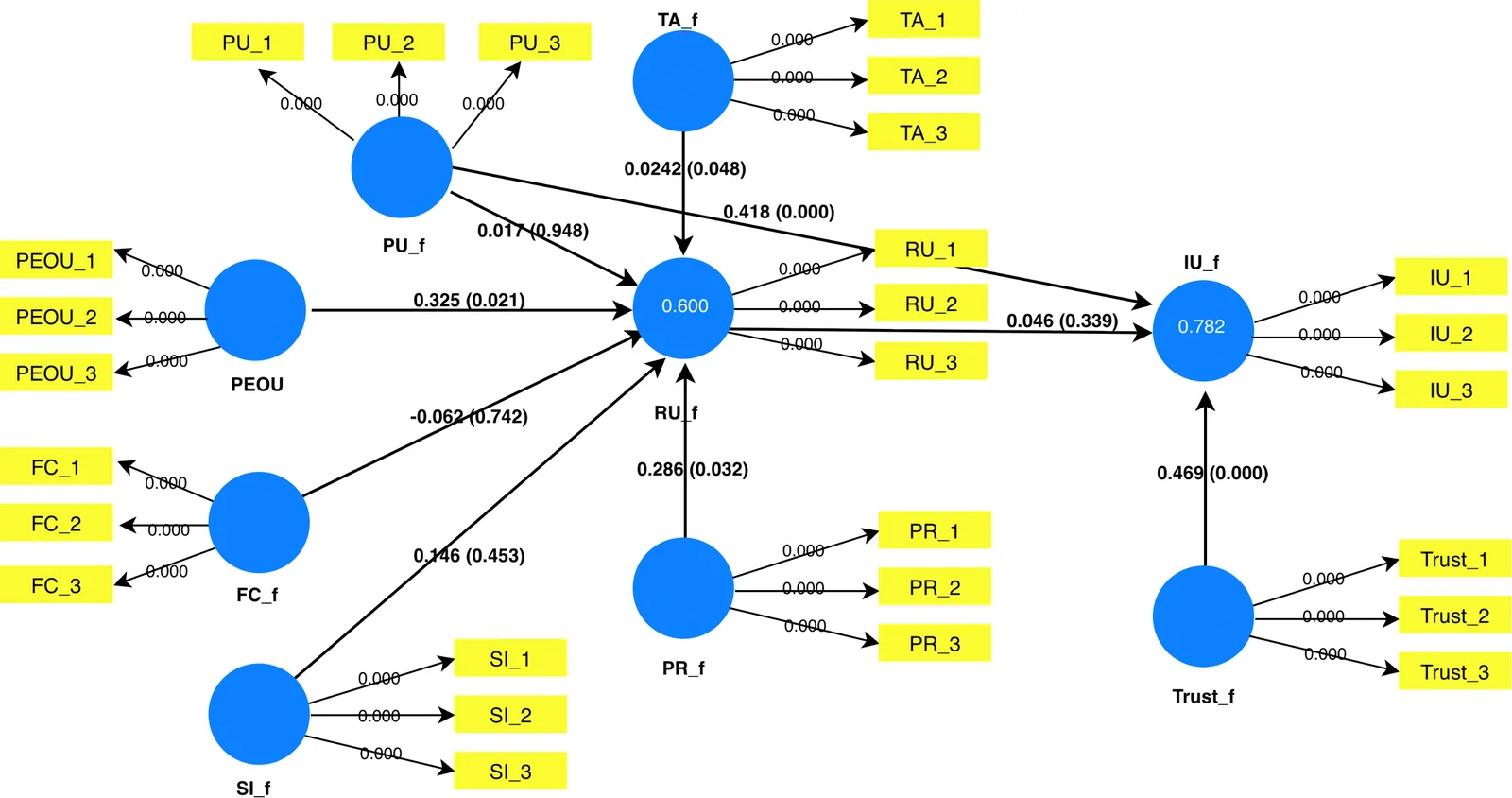
Result of SmartPLS. Source: Author’s Work.

Table 4 illustrates the result of hypothesis testing to indicate the relationship among the factors in the study using the bootstrapping feature in SmartPLS. The result shows that the proposed hypotheses are supported, indicating the positive relationship between dependent and independent variables except for PU and RU (H1), FC and RU (H5), SI and RU (H6), and RU and IU (H8) in Table 4.

**Table 4:** Hypothesis test result.

Hypothesis	Path	Standardized path coefficient	p-Values	Results
H: 1	PU > RU	0.017	0.948	Rejected
H: 2	PEOU > RU	0.325	0.021	Supported
H: 3	TA > RU	0.242	0.048	Supported
H: 4	PU > IU	0.418	0.000	Supported
H: 5	FC > RU	−0.062	0.742	Rejected
H: 6	SI > RU	0.146	0.453	Rejected
H: 7	PR > RU	0.286	0.032	Supported
H: 8	RU > IU	0.046	0.339	Rejected
H: 9	Trust > IU	0.469	0.000	Supported

## Discussion

The findings of this study contribute to valuable insight into the drivers and barriers to the adoption and acceptance of telemedicine in post-pandemic Bangladesh, based on two of the most powerful models of technology diffusion: TAM and UTAUT2. It was found that some highly critical determinants of behavioral intention were trust and perceived usefulness, while perceived risk and technology anxiety were also raised as concerns. From the results, it can be seen that trust and perceived usefulness are the most important factors determining a patient’s intention to use telemedicine. It is in agreement with earlier studies that had showed users were more likely to adopt the use of telemedicine services, especially when the feeling of trust in technology and perceived clear benefits are there for convenience, access to specialists, or improved healthcare delivery [32,68,69]. Therefore, telemedicine reinforces perceived value due to its potential to reduce travel time, enhance continuity of care, and provide access to specialists otherwise unavailable [9].

The study also found that perceived ease of use was a significant predictor of resistance to use. Research findings on telemedicine adoption indicate that users are more likely to embrace telemedicine platforms if they see them as user-friendly and intuitive [46,49]. In post-pandemic Bangladesh, with the fact that the large portion of the population may still be relatively new to digital health platforms, ease of use becomes paramount. In this regard, it follows that healthcare professionals and developers alike must turn their attention to the development of interfaces which make such barriers as minimal as possible, especially in cases where elderly patients or those not in possession of high digital literacy are concerned. A number of studies on telemedicine service interfaces also indicate that future telemedicine platforms should be designed with a focus on user-friendliness, particularly for elderly people [70–73]. However, one of the interesting findings of this study is that resistance to use and behavioral intention were not found statistically significant. This goes against the conventional thinking that resistance-especially with regard to new technologies-creates a barrier very important to adoption [19,46]. This in turn may be an effect of the COVID-19 pandemic, which fostered a feeling of urgency for modes of healthcare delivery that were an alternative to traditional face-to-face care. In Bangladesh, the pandemic might have reduced resistance to the adoption of telemedicine on the part of users, out of necessity, when they previously would have had many misgivings.

On the other hand, perceived risk and technology anxiety contributed significantly to the resistance to use respectively. Study conducted in China and Pakistan also revealed that perceived risk and technology anxiety negatively influence behavioral intentions towards telemedicine services [32,49,74]. This again complements and supports most of the global literature by citing the need to address security concerns in using the mobile health services, especially where data about patients and their privacy are involved [8]. In countries like Bangladesh, which are still in the development stage regarding digital literacy and measures related to cybersecurity, people are still afraid of data breaches or misuse of sensitive information; hence, this is an issue and acts as the major barrier to the adoption of telemedicine. The future strategy for increasing the usage of telemedicine must focus on enhancing the security infrastructure of these platforms and effectively communicating these safeguards to users [32,33,74]. Although trust and security being of utmost importance, facilitating conditions and social influence were not significantly related to resistance to use. Thus, this implies that just because one has access to the required technology or is influenced by peers might not serve as motivation enough for the users to adopt telemedicine. Instead, perceived usefulness of the telemedicine services and trust in the system come first. In other words, healthcare providers should ensure not only that the technology is available but also that they clearly communicate exactly how it is useful to patients in Bangladesh.

The social influence, which commonly plays a part in the adoption of technologies, may be less strong in the context of healthcare in Bangladesh. This is maybe for cultural reasons; whereby individual health decisions rely more on healthcare providers than those of peers or societal pressures [75,76]. This insight is very important herewith because it goes to underscore the need for more emphasis on patient education campaigns led by healthcare professionals and less reliance on social influence as an engine of adoption.

### Limitations and future directions

This study has some limitations. First, the cross-sectional design limits causal inference between the constructs. Second, the use of convenience and snowball sampling may reduce generalizability to all telemedicine users in Bangladesh. Third, self-reported data may be affected by response bias. Future research should use longitudinal or mixed-method designs, probability sampling, and broader geographic coverage to improve external validity and examine changes in telemedicine adoption over time.

## Conclusion

The present study clearly establishes trust, perceived usefulness, and ease of use as significant drivers for the adoption of telemedicine services in Bangladesh. The direct path between resistance to use and behavioural intention was not significant in this model, but perceived risk and technology anxiety still play very important roles in shaping resistance to telemedicine services. Dealing with such concerns, especially by introducing better cybersecurity and educating users, becomes quite critical for the future of telemedicine services in Bangladesh.

To actualize the true potential of telemedicine, stakeholders must work together to develop secure and user-friendly platforms while making aware both professionals in health and patients regarding the benefits of such services. Facilitating conditions and social influence were not significant predictors of resistance to use in this model, but they should not be dismissed, as they may still shape adoption indirectly by supporting an enabling environment that encourages telemedicine use. Addressing the identified key factors in this study will, therefore, go a long way toward improving the adoption of telemedicine by policymakers and healthcare providers in Bangladesh in ensuring this valuable health service reaches the needy, especially in rural and underserved areas.

## References

[1] Statista. Internet and social media users in the world 2024. In: Statista [Internet]. 2024 [cited 2024 Nov 23]. Available from: https://www.statista.com/statistics/617136/digital-population-worldwide/

[2] DataReportal. Digital 2024: Bangladesh. In: DataReportal – Global Digital Insights [Internet]. 2024 [cited 2024 Nov 23]. Available from: https://datareportal.com/reports/digital-2024-bangladesh

[3] Byun H, Park J. A Study on the Intention to Use Korean Telemedicine Services: Focusing on the UTAUT2 Model. In: Kim J, Lee R, editors. Data Science and Digital Transformation in the Fourth Industrial Revolution. Cham: Springer International Publishing; 2021. pp. 1–12. doi: 10.1007/978-3-030-64769-8_1

[4] Macrotrends. Bangladesh Rural Population 1960-2024. 2024 [cited 2024 Nov 23]. Available from: https://www.macrotrends.net/global-metrics/countries/bgd/bangladesh/rural-population

[5] Walters J, Johnson T, DeBlasio D, Klein M, Sikora K, Reilly K, et al. Integration and impact of telemedicine in underserved pediatric primary care. Clin Pediatr (Phila). 2021;60(11–12):452–8. doi: 10.1177/00099228211039621 34382880

[6] Zobair K, Sanzogni L, Sandhu K. Telemedicine healthcare service adoption barriers in rural Bangladesh. Australas J Inform Syst. 2020;24. doi: 10.3127/ajis.v24i0.2165

[7] Gupta A, Dogar ME, Zhai ES, Singla P, Shahid T, Yildirim HN, et al. Innovative telemedicine approaches in different countries: opportunity for adoption, leveraging, and scaling-up. Telehealth Med Today. 2019;5. doi: 10.30953/tmt.v5.160

[8] Chowdhury SR, Sunna TC, Ahmed S. Telemedicine is an important aspect of healthcare services amid COVID-19 outbreak: Its barriers in Bangladesh and strategies to overcome. Int J Health Plann Manage. 2021;36(1):4–12. doi: 10.1002/hpm.3064 32857887

[9] Deldar K, Bahaadinbeigy K, Tara SM. Teleconsultation and clinical decision making: A systematic review. Acta Inform Med. 2016;24(4):286–92. doi: 10.5455/aim.2016.24.286-292 27708494 PMC5037984

[10] Baudier P, Kondrateva G, Ammi C, Chang V, Schiavone F. Patients’ perceptions of teleconsultation during COVID-19: A cross-national study. Technol Forecast Soc Change. 2021;163:120510. doi: 10.1016/j.techfore.2020.120510 33318716 PMC7720768

[11] Martins NLM, Duarte P, Pinho JCMR. An analysis of determinants of the adoption of mobile health (mhealth). Rev Adm Empres. 2021;61:e2019–0239. doi: 10.1590/S0034-759020210403x

[12] Melinda T, Setiawati CI. Analisis minat pengguna layanan telemedicine Halodoc di kota Bandung dengan menggunakan model modifikasi UTAUT2. SEIKO J Manag Bus. 2022;5:262–73. doi: 10.37531/sejaman.v5i2.2212

[13] Suroso JS, Sukmoro TC. Factors affecting behavior of the use of healthcare mobile application technology in Indonesian society. J Theor Appl Inf Technol. 2021;99:3923–34.

[14] Aggelidis VP, Chatzoglou PD. Using a modified technology acceptance model in hospitals. Int J Med Inform. 2009;78(2):115–26. doi: 10.1016/j.ijmedinf.2008.06.006 18675583

[15] Rouidi M, Elouadi AE, Hamdoune A, Choujtani K, Chati A. TAM-UTAUT and the acceptance of remote healthcare technologies by healthcare professionals: A systematic review. Inform Med Unlocked. 2022;32:101008. doi: 10.1016/j.imu.2022.101008

[16] Davis FD. Perceived Usefulness, perceived ease of use, and user acceptance of information technology. MIS Q. 1989;13(3):319–40. doi: 10.2307/249008

[17] An MH, You SC, Park RW, Lee S. Using an extended technology acceptance model to understand the factors influencing telehealth utilization after flattening the COVID-19 curve in South Korea: Cross-sectional survey study. JMIR Med Inform. 2021;9(1):e25435. doi: 10.2196/25435 33395397 PMC7801132

[18] Venkatesh V, Morris MG, Davis GB, Davis FD. User acceptance of information technology: Toward a unified view. MIS Q. 2003;27(3):425–78. doi: 10.2307/30036540

[19] Hoque R, Sorwar G. Understanding factors influencing the adoption of mHealth by the elderly: An extension of the UTAUT model. Int J Med Inform. 2017;101:75–84. doi: 10.1016/j.ijmedinf.2017.02.002 28347450

[20] Shiferaw KB, Mengiste SA, Gullslett MK, Zeleke AA, Tilahun B, Tebeje T, et al. Healthcare providers’ acceptance of telemedicine and preference of modalities during COVID-19 pandemics in a low-resource setting: An extended UTAUT model. PLoS One. 2021;16(4):e0250220. doi: 10.1371/journal.pone.0250220 33886625 PMC8061916

[21] Nassuora AB. Students acceptance of mobile learning for higher education in Saudi Arabia. Int J Learn Manag Syst. 2013;1:1–9. doi: 10.18576/ijlms/010101

[22] Ammenwerth E. Technology acceptance models in health informatics: TAM and UTAUT. Stud Health Technol Inform. 2019;263:64–71. doi: 10.3233/SHTI190111 31411153

[23] Venkatesh V, Thong JYL, Xu X. Consumer acceptance and use of information technology: Extending the unified theory of acceptance and use of technology. MIS Q. 2012;36(1):157–78. doi: 10.2307/41410412

[24] Rahi S. Assessing individual behavior towards adoption of telemedicine application during COVID-19 pandemic: evidence from emerging market. Libr Hi Tech. 2021;40(2):394–420. doi: 10.1108/lht-01-2021-0030

[25] Octavius GS, Antonio F. Antecedents of intention to adopt mobile health (mHealth) Application and its impact on intention to recommend: An evidence from Indonesian customers. Int J Telemed Appl. 2021;2021:6698627. doi: 10.1155/2021/6698627 34012467 PMC8105118

[26] Uddin KMS, Bhuiyan MRI, Hamid M. Perception towards the acceptance of digital health services among the people of Bangladesh. WSEAS Trans Bus Econ. 2024;21:1557–70. doi: 10.37394/23207.2024.21.127

[27] Duarte P, Pinho JC. A mixed methods UTAUT2-based approach to assess mobile health adoption. J Bus Res. 2019;102:140–50. doi: 10.1016/j.jbusres.2019.05.022

[28] Wu C, Lim GG. Investigating older adults users’ willingness to adopt wearable devices by integrating the technology acceptance model (UTAUT2) and the Technology Readiness Index theory. Front Public Health. 2024;12:1449594. doi: 10.3389/fpubh.2024.1449594 39421816 PMC11484832

[29] Soren AA, Chakraborty S. Adoption, satisfaction, trust, and commitment of over-the-top platforms: An integrated approach. J Retail Consum Serv. 2024;76:103574. doi: 10.1016/j.jretconser.2023.103574

[30] Adenuga KI, Iahad NA, Miskon S. Towards reinforcing telemedicine adoption amongst clinicians in Nigeria. Int J Med Inform. 2017;104:84–96. doi: 10.1016/j.ijmedinf.2017.05.008 28599820

[31] Shadangi P, Kar S, Mohanty AK, Dash M. Physician’s attitude towards acceptance of telemedicine technology for delivering health care services. Int J Mech Eng Technol. 2018;9:715–22.

[32] Kamal SA, Shafiq M, Kakria P. Investigating acceptance of telemedicine services through an extended technology acceptance model (TAM). Technol Soc. 2020;60:101212. doi: 10.1016/j.techsoc.2019.101212

[33] Featherman MS, Pavlou P. Predicting e-services adoption: A perceived risk facets perspective. Int J Hum Comput Stud. 2003;59:451–74. doi: 10.1016/S1071-5819(03)00111-3

[34] Lee W-I, Fu H-P, Mendoza N, Liu T-Y. Determinants impacting user behavior towards emergency use intentions of m-health services in Taiwan. Healthcare (Basel). 2021;9(5):535. doi: 10.3390/healthcare9050535 34063637 PMC8147645

[35] Kalinić Z, Marinković V, Djordjevic A, Liebana-Cabanillas F. What drives customer satisfaction and word of mouth in mobile commerce services? A UTAUT2-based analytical approach. J Enterp Inf Manag. 2020;33:71–94. doi: 10.1108/JEIM-05-2019-0136

[36] Parasuraman A. Technology readiness index (Tri): A multiple-item scale to measure readiness to embrace new technologies. J Serv Res. 2000;2:307–20. doi: 10.1177/109467050024001

[37] Cambre MA, Cook DL. Computer anxiety: Definition, measurement, and correlates. J Educ Comput Res. 1985;1:37–54. doi: 10.2190/FK5L-092H-T6YB-PYBA

[38] Thong JYL, Hong S-J, Tam KY. The effects of post-adoption beliefs on the expectation-confirmation model for information technology continuance. Int J Hum Comput Stud. 2006;64:799–810. doi: 10.1016/j.ijhcs.2006.05.001

[39] Brown SA, Venkatesh V. Model of adoption of technology in households: A baseline model test and extension incorporating household life cycle. MIS Q. 2005;29(3):399–426. doi: 10.2307/25148690

[40] Cimperman M, Makovec Brenčič M, Trkman P. Analyzing older users’ home telehealth services acceptance behavior-applying an Extended UTAUT model. Int J Med Inform. 2016;90:22–31. doi: 10.1016/j.ijmedinf.2016.03.002 27103194

[41] Beyari H, Abareshi A. An Empirical Study of How Social Influence Impacts Customer Satisfaction with Social Commerce Sites. In: Saeed F, Gazem N, Mohammed F, Busalim A, editors. Recent Trends in Data Science and Soft Computing. Cham: Springer International Publishing; 2019. pp. 973–984. doi: 10.1007/978-3-319-99007-1_90

[42] Viswanathan P, Singh AB, Gupta G. The role of social influence and e-service quality in impacting loyalty for online life insurance: A SEM-based study. Int J Bus Excell. 2020;20:322–37. doi: 10.1504/IJBEX.2020.106370

[43] Yang M, Jiang J, Kiang M, Yuan F. Re-examining the impact of multidimensional trust on patients’ online medical consultation service continuance decision. Inf Syst Front. 2022;24(3):983–1007. doi: 10.1007/s10796-021-10117-9 33688300 PMC7932182

[44] Mou J, Shin D-H, Cohen J. Health beliefs and the valence framework in health information seeking behaviors. Inf Technol People. 2016;29(4):876–900. doi: 10.1108/itp-06-2015-0140

[45] Guo X, Zhang X, Sun Y. The privacy–personalization paradox in mHealth services acceptance of different age groups. Electron Commer Res Appl. 2016;16:55–65. doi: 10.1016/j.elerap.2015.11.001

[46] Ludeña-Poma J, Rojas-Gallegos S, Solano-Ortiz L, Castro-Chavez Y, Chahua-Borda D, Ludeña-Poma J. Influence of resistance to use, perceived ease of use, perceived usefulness, and facilitating conditions in the intention to use telemedicine in Peru. Pharmacia. 2024;71:1–11. doi: 10.3897/pharmacia.71.e118004

[47] Russell S. Treatment-seeking behaviour in urban Sri Lanka: trusting the state, trusting private providers. Soc Sci Med. 2005;61(7):1396–407. doi: 10.1016/j.socscimed.2004.11.077 16005775

[48] Mohseni M, Lindstrom M. Social capital, trust in the health-care system and self-rated health: The role of access to health care in a population-based study. Soc Sci Med. 2007;64(7):1373–83. doi: 10.1016/j.socscimed.2006.11.023 17202025

[49] Zhu L, Jiang X, Cao J. Factors affecting continuance intention in non-face-to-face telemedicine services: trust typology and privacy concern perspectives. Healthcare (Basel). 2023;11(3):374. doi: 10.3390/healthcare11030374 36766949 PMC9914105

[50] Abbad MMM. Using the UTAUT model to understand students’ usage of e-learning systems in developing countries. Educ Inf Technol (Dordr). 2021;26(6):7205–24. doi: 10.1007/s10639-021-10573-5 34025204 PMC8122219

[51] Abbas RM, Carroll N, Richardson I. In Technology We Trust: Extending TAM from a Healthcare Technology Perspective. 2018 IEEE International Conference on Healthcare Informatics (ICHI). 2018. pp. 348–9. doi: 10.1109/ICHI.2018.00051

[52] Raitoharju R. When Acceptance is not Enough - Taking TAM-Model into Healthcare. Proceedings of the 38th Annual Hawaii International Conference on System Sciences. 2005. pp. 150c. doi: 10.1109/HICSS.2005.691

[53] Rochadiat AMP, Tong ST, Hancock JT, Stuart-Ulin CR. The outsourcing of online dating: Investigating the lived experiences of online dating assistants working in the contemporary gig economy. Social Media + Society. 2020;6(3). doi: 10.1177/2056305120957290

[54] Sonenstein FL. Introducing the well-being of adolescents in vulnerable environments study: Methods and findings. J Adolesc Health. 2014;55(6 Suppl):S1–3. doi: 10.1016/j.jadohealth.2014.09.008 25453997

[55] Raifman S, DeVost MA, Digitale JC, Chen YH, Morris MD. Respondent-driven sampling: A sampling method for hard-to-reach populations and beyond. Curr Epidemiol Rep. 2022;9:38–47. doi: 10.1007/s40471-022-00287-8

[56] Muhib FB, Lin LS, Stueve A, Miller RL, Ford WL, Johnson WD, et al. A venue-based method for sampling hard-to-reach populations. Public Health Rep. 2001;116 Suppl 1(Suppl 1):216–22. doi: 10.1093/phr/116.S1.216 11889287 PMC1913675

[57] Magnani R, Sabin K, Saidel T, Heckathorn D. Review of sampling hard-to-reach and hidden populations for HIV surveillance. AIDS. 2005;19 Suppl 2:S67–72. doi: 10.1097/01.aids.0000172879.20628.e1 15930843

[58] Ammenwerth E, Hackl WO. Topics for Continuous Education in Nursing Informatics: Results of a Survey Among 280 Austrian Nurses. dHealth 2019 – From eHealth to dHealth. IOS Press; 2019. pp. 162–9. doi: 10.3233/978-1-61499-971-3-162 31118333

[59] Berry L, Walter J, Johnson J, Alton J, Powers J, Llòria X, et al. Patient-reported experience with Fabry disease and its management in the real-world setting: results from a double-blind, cross-sectional survey of 280 respondents. Orphanet J Rare Dis. 2024;19(1):153. doi: 10.1186/s13023-024-03090-4 38605390 PMC11007961

[60] Rathakrishnan B, Singh SSB, Yahaya A, Kamaluddin MR, Aziz SFA. The relationship among spirituality, fear, and mental health on COVID-19 among adults: An exploratory research. Front Psychol. 2022;12:815332. doi: 10.3389/fpsyg.2021.815332 PMC8790181 35095699

[61] Ariffin SK, Ihsannuddin NQ, Mohsin AMA. The influence of attitude functions on Muslim consumer attitude towards social media advertising: A case of bubble tea. J Islam Mark. 2021;13:2308–30. doi: 10.1108/JIMA-01-2021-0015

[62] Apanga PA, Adam MA. Factors influencing the uptake of family planning services in the Talensi District, Ghana. Pan Afr Med J. 2015;20:10. doi: 10.11604/pamj.2015.20.10.5301 25995807 PMC4430143

[63] Waqas A, Khan S, Sharif W, Khalid U, Ali A. Association of academic stress with sleeping difficulties in medical students of a Pakistani medical school: A cross sectional survey. PeerJ. 2015;3:e840. doi: 10.7717/peerj.840 25802809 PMC4369327

[64] Hair JF, Astrachan CB, Moisescu OI, Radomir L, Sarstedt M, Vaithilingam S. Executing and interpreting applications of PLS-SEM: Updates for family business researchers. J Fam Bus Strateg. 2021;12:100392. doi: 10.1016/j.jfbs.2020.100392

[65] Nunnally JC. An Overview of Psychological Measurement. In: Wolman BB, editor. Clinical Diagnosis of Mental Disorders: A Handbook. Boston, MA: Springer US; 1978. pp. 97–146. doi: 10.1007/978-1-4684-2490-4_4

[66] Hair JF, Anderson RE, Tatham RL. Multivariate data analysis with readings. 2nd ed. New York: Macmillan; 1986.

[67] Hair J, Alamer A. Partial Least Squares Structural Equation Modeling (PLS-SEM) in second language and education research: Guidelines using an applied example. Res Methods Appl Linguist. 2022;1(3):100027. doi: 10.1016/j.rmal.2022.100027

[68] Bashshur RL, Howell JD, Krupinski EA, Harms KM, Bashshur N, Doarn CR. The empirical foundations of telemedicine interventions in primary care. Telemed J E Health. 2016;22(5):342–75. doi: 10.1089/tmj.2016.0045 27128779 PMC4860623

[69] Almathami HKY, Win KT, Vlahu-Gjorgievska E. Barriers and facilitators that influence telemedicine-based, real-time, online consultation at patients’ homes: Systematic literature review. J Med Internet Res. 2020;22(2):e16407. doi: 10.2196/16407 32130131 PMC7059083

[70] Attila A, Garai A, Pentek I. Common open telemedicine hub and infrastructure with interface recommendation. 2016 IEEE 11th International Symposium on Applied Computational Intelligence and Informatics (SACI). Timisoara, Romania: IEEE; 2016. pp. 385–90. doi: 10.1109/saci.2016.7507407

[71] Chuna YJ, Patterson PE. A suggestion for future research on interface design of an Internet-based telemedicine system for the elderly. Work. 2012;41 Suppl 1:353–6. doi: 10.3233/WOR-2012-0181-353 22316748

[72] Choi D-J. The role and prospects of telemedicine in the treatment of heart failure patients: A narrative review. Ewha Med J. 2025;48(2):e26. doi: 10.12771/emj.2025.00360 40703378 PMC12277493

[73] Vasquez-Cevallos L, Mitchell A, Muñoz-Hernández S, Herranz-Nieva Á, Garcia-Mingo A, De Corral-San Martin P. Educational and clinical applications of a web- and Android-based telemedicine platform to expand rural health care in Ecuador. Telemed Rep. 2025;6:67–75. doi: 10.1089/tmr.2024.0091 PMC11947644 40151789

[74] Liu C, Wang J, Chen R, Zhou W. Exploring the influence of Chinese online patient trust on telemedicine behavior: Insights into perceived risk and behavior intention. Front Public Health. 2024;12:1415889. doi: 10.3389/fpubh.2024.1415889 39247232 PMC11377225

[75] Rumi MH, Makhdum N, Rashid MH, Muyeed A. Gender differences in service quality of Upazila Health Complex in Bangladesh. J Patient Exp. 2021;8. doi: 10.1177/23743735211008304 34179433 PMC8205356

[76] Rumi MH, Makhdum N, Rashid MH, Muyeed A. Patients’ satisfaction on the service quality of Upazila Health Complex in Bangladesh. J Patient Exp. 2021;8. doi: 10.1177/23743735211034054 34395848 PMC8361520

