# Supplementary material for: Understanding the influencing factors of telemedicine service adoption in post-pandemic Bangladesh: An extended TAM and UTAUT2 model analysis
Source: PLoS One. 2026 Oct 28;21(10):e0361691. doi: 10.1371/journal.pone.0361691 (PMC13716190; doi:10.1371/journal.pone.0361691)
Supplement: Supplementary file 2 [file pone.0361691.s003.docx]

# Author Response Letter

25 August, 2025

Dear Editor and Reviewers,

We are grateful for the reviewers’ constructive feedback. All comments have been carefully addressed, and revisions have been incorporated to improve clarity, accuracy, and presentation of the manuscript. We believe the manuscript number PONE-D-24-59687R1which entitles “**Understanding the Influencing Factors of Telemedicine Service Adoption in Post-Pandemic Bangladesh: An Extended TAM and UTAUT2 Model Analysis**” is now suitable for publication and hope it will be considered for acceptance.

We sincerely thank the reviewers for their constructive comments and valuable feedback. We have carefully revised the manuscript to address all the points raised. Below, we provide a detailed, point-by-point response to each reviewer’s comments.

| **Reviewer 1 Comment and Author Responses** | |
| --- | --- |
| **Reviewer 1 Comments** | **Author Response** |
| Comment #1: All comments have been addressed | We would like to extend our sincere appreciation to the reviewers for their valuable time, insightful comment. We appreciate the reviewer’s acknowledgment. |
| Comment #2: The manuscript currently has a heading titled "Discussion and Conclusion", followed by a separate heading "Conclusion". To maintain clarity, it is suggested to use the heading "Discussion" only. | We thank the reviewer for this suggestion. We have revised the section heading to "Discussion" only, and retained a separate "Conclusion" section at the end for clarity and consistency. |
| **Reviewer 2 Comment and Author Responses** | |
| **Reviewer 2 Comments** | **Author Response** |
| Comment #1: There are spelling errors in some parts of the text (e.g., Line 37 and Line 317). | We thank the reviewer for this suggestion. We have thoroughly proofread the manuscript and corrected the spelling errors throughout. |
| Comment #2: Please mention keywords based on MeSH. | We would like to extend our sincere appreciation to the reviewers for their valuable time, insightful comment. We have revised the keywords according to the MeSH terms.  **Keywords:** Telemedicine, Technology Acceptance, Post-Pandemic, Attitude to Health, Cross-Sectional Studies, Bangladesh |
| Comment #3: According to the submission guidelines, abbreviate the names of the journals in the references. | We are grateful for the reviewers’ constructive feedback. We have reformatted the reference list to use abbreviated journal names as per the submission guidelines. |
| Comment #4: Please correct the partial disorganizations in Figure 2. | Figure 2 has been reformatted and corrected for clarity and proper alignment. Thank you so much. |
| Comment #5: Please include the abbreviation of each word where it is first mentioned (e.g., Line 93, Line 95, Line 30, Line 122). | We would like to extend our sincere appreciation to the reviewers for their valuable time, insightful comment. We have ensured that all abbreviations are defined at their first occurrence in the manuscript. |
| Comment #6: You have mentioned the conclusion section separately. Therefore, it would be better to remove the word “conclusion” from the title of the discussion section (Line 367). | We would like to extend our sincere appreciation to the reviewers for their valuable time, insightful comment. As suggested, we have modified the heading to 'Discussion' only. The Conclusion remains as a separate final section. |
| Comment #7: Did you have any limitations in this study? | We have added a Limitations subsection in the Discussion section to acknowledge the study’s limitations. We are grateful for the reviewers’ constructive feedback. |
| Comment #8: Please describe each table before citing it. | We have revised the text to provide a descriptive introduction for each table before referencing it. |
| Comment #9: In Lines 325–326, it is stated that around 88% of respondents have internet access in Table 2, but this result appears in Table 1. Please correct. | We apologize for this oversight. The error has been corrected, and the description now refers to the correct table. We would like to extend our sincere appreciation to the reviewers for their valuable time, insightful comment. |
| Comment #10: Please write the number of tables and figures in the same format (Latin I, II, III… or modern 1, 2, 3…). | We have standardized the numbering format for tables and figures to Latin I, II, III. We are grateful for the reviewers’ constructive feedback. |
| Comment #11: Please paraphrase the paragraph after Figure 2 (Lines 347–359). | The paragraph after Figure 2 has been paraphrased and revised for better readability and originality. Thank you so much. |
