# Supplementary material for: Understanding the influencing factors of telemedicine service adoption in post-pandemic Bangladesh: An extended TAM and UTAUT2 model analysis
Source: PLoS One. 2026 Oct 28;21(10):e0361691. doi: 10.1371/journal.pone.0361691 (PMC13716190; doi:10.1371/journal.pone.0361691)
Supplement: Supplementary file 3 [file pone.0361691.s002.docx]

Dear Editor and Reviewers,

I hope you all are doing well. Thank you for your efforts in reviewing our manuscript. I hope your valuable feedback will enrich our paper. Here are the responses from the authors to your feedback.

**Reviewer #1:**

Dear Author,

I have reviewed your manuscript, and the topic is highly relevant in the post-COVID era. However, several key aspects require further refinement to enhance its suitability for publication. Below are my detailed comments and recommendations:

1. Introduction: In line 89, consider incorporating examples of telemedicine adoption in different countries before presenting the rationale for focusing on Bangladesh. This will strengthen the study's contextual justification.

**Response:**

Thank for your feedback. I think this will add value to our paper and we have incorporated some examples of telemedicine adoption in different countries before presenting the rationale for focusing on Bangladesh.

2. Sampling Technique: Provide details on the study area and the methodology used to determine the sample size of 276 respondents. You may refer to this article for guidance on sampling techniques, study population, and study area: PLOS ONE Article.

**Response:**

Thank for honorable reviewer. Though you haven’t received the mentioned guiding article, however we chose it as many researchers used same number of respondents to determine different factors and health issues; we have mentioned some of those papers.

3. Socio-Demographic Table: The greater than (>) and less than (<) signs in the "Age of Respondents" and "Monthly Income" sections appear to be misplaced. For instance, "> 20" should likely be "< 20," and "< 50" should be "> 50." Similarly, "> 10000" should be "< 10000," and "< 50000" should be "> 50000." Please verify and correct these inconsistencies.

**Response:**

Thank for the valuable feedback. We have corrected these inconsistencies.

4. Discussion Section: Strengthen the discussion by comparing your hypothesis findings with those of similar studies to highlight key similarities or differences.

**Response:**

Thank for the valuable feedback. We have added some comparison of my findings with similar studies.

5. Referencing: Ensure all citations and references follow the APA 7 format for consistency and accuracy.

**Response:**

Thank for the valuable feedback. We reviewed all the citations.

6. Sampling Justification: The explanation of the sampling technique lacks clarity. Elaborate on the rationale behind the chosen approach and justify its appropriateness for the study.

**Response:**

Thank for the valuable feedback. Rationale behind the chosen approach and justification has been added.

7. Structural Equation Modeling (SEM): Many tables and figures related to SEM analysis are either insufficiently analyzed or not clearly presented. Please ensure that all relevant statistical outputs are appropriately included.

**Response:**

Thank for your feedback. We have reviewed and tried to identify those. However it seems to us perfect and we added some articles from renowned publishers those we followed while analyzing our data. These are.

<https://doi.org/10.1016/j.techsoc.2019.101212>

<https://doi.org/10.1186/s12911-022-01917-3>

[https://doi.org/10.1186/s13731‑023‑00336‑6](https://doi.org/10.1186/s13731023003366)

8. Robustness Checks: Key methodological concerns such as endogeneity, unobserved heterogeneity, and linearity assessments appear to be overlooked. Consider conducting these robustness checks to validate your findings.

**Response:**

Thank you for your insightful feedback. While we recognize the importance of robustness checks, we think current methodology and model align with the research objectives and context, and we believe they adequately address the research questions. We appreciate your suggestions and will consider them for future studies.

9. Theoretical Framework: The integration of TAM and UTAUT2 is not well articulated. Provide a detailed discussion on how these models complement each other and justify their combined use in the study.

**Response:**

Thank you for your feedback. Discussion on how these models complement each other and justification of their combined use in the study has been added.

10. Data Collection Approach: The use of semi-structured interviews to collect Likert scale data is unclear. Please clarify the rationale behind this methodological choice.

**Response:**

Thank you for your insightful feedback. We apologize for mentioning interviews, it will be semi-structured survey questionnaire.

11. Methodology: The overall methodology section requires significant improvement in terms of clarity, rigor, and justification. Ensure that each methodological step is thoroughly explained.

**Response:**

Thank you for your insightful feedback. We have updated accordingly.

12. General Structure: Refer to well-developed published papers for guidance on structuring the manuscript effectively. You may follow this paper: PLOS ONE Article.

**Response:**

Thank you for your insightful feedback. Though there is no link of particular paper, however, we have followed the sample paper link provided by the Editor.

With these revisions and improvements, I believe the paper will be in a stronger position for publication.

Thank you for your kind words. We hope publishing this paper will provide insights to the policy maker of our country as well as will motivate us to conduct more research.
