# Supplementary material for: Understanding the influencing factors of telemedicine service adoption in post-pandemic Bangladesh: An extended TAM and UTAUT2 model analysis
Source: PLoS One. 2026 Oct 28;21(10):e0361691. doi: 10.1371/journal.pone.0361691 (PMC13716190; doi:10.1371/journal.pone.0361691)
Supplement: Supplementary file 4 [file pone.0361691.s004.docx]

**Response to Reviewers**

**Reviewer 2**

| **Comments** | | **Authors’ Responses** |
| --- | --- | --- |
| Some comments were not addressed: Comments 1, 2, 3, 5, 7, 8, and 10! These comments are not suggestions and are provided to improve your paper. Please investigate them carefully again. | | We thank the reviewer for flagging these. We have re-reviewed the manuscript carefully and confirmed/completed the following revisions (all marked as tracked changes in the resubmitted file). |
| Comment 1 | There are spelling errors in some parts of the text (for example, Line 37 and Line 317). Please reread the text and correct the spelling errors. | We have proofread the manuscript again and corrected the spelling and grammatical errors identified, including in the Abstract and Discussion sections. Additional minor corrections (e.g., "finding highpoints" to "findings highlight", "research's" to "research has been", "believes" to "beliefs", "furthermost established" to "most well-established", duplicated "mobile mobile", "For examples" to "For example", subject-verb agreement in "Telemedical services covers") have also been corrected throughout the manuscript. All changes are tracked. |
| Comment 2 | Please mention keywords based on MeSH. | The keywords have been revised to MeSH-based terms: Telemedicine; Patient Acceptance of Health Care; Health Services Accessibility; Attitude to Health; Cross-Sectional Studies; Bangladesh. |
| Comment 3 | According to the submission guidelines, please abbreviate the “names of the journals” in the references. | All references have been reviewed and journal names abbreviated according to standard NLM/PubMed style, consistent with PLOS ONE submission guidelines (e.g., "Technological Forecasting and Social Change" to "Technol Forecast Soc Change"; "MIS Quarterly" to "MIS Q"; "Journal of Business Research" to "J Bus Res"; "Journal of Retailing and Consumer Services" to "J Retail Consum Serv"; "Technology in Society" to "Technol Soc"; "International Journal of Telemedicine and Applications" to "Int J Telemed Appl"; "International Journal of Medical Informatics" to "Int J Med Inform"). |
| Comment 5 | Please include the abbreviation of each word where it is first mentioned (For example: line 93 and line 95, or line 30 and line 122). | We have re-audited every abbreviation in the manuscript. Two abbreviations used without a prior full-form definition have now been corrected: "AVE" is now introduced as "Average Variance Extracted (AVE)" and "HTMT" as "Heterotrait-Monotrait ratio (HTMT)" at their first mention in the Results section (previously HTMT was only spelled out in the Table III caption, which appeared after its first use in the text). All other abbreviations (TAM, UTAUT2, PU, PEOU, TA, RU, FC, SI, PR, IU, TLS, PLS-SEM, mHealth) were checked and confirmed to be defined in full at their first occurrence in the text. |
| Comment 7 | Did you have any limitations in this study? | Yes. A "Limitations and Future Directions" section is included before the Conclusion, noting the cross-sectional design, convenience/snowball sampling, and self-report bias as limitations, with corresponding suggestions for future research. |
| Comment 8 | Please describe each table before citing it. | A brief lead-in sentence introducing and describing the content of each table (Table I-IV) has been added immediately before the table appears, so that each table is described before it is cited/presented. |
| Comment 10 | Please write the number of tables and figures in the same format (Latin format (I, II, III, ..) or modern format (1, 2, 3, ..). | Figure numbering has been changed from Arabic (1, 2) to Roman numerals (I, II) so that it is consistent with the Table numbering format (Table I-IV), as requested. |

**Reviewer 3**

| **Comments** | **Authors’ Responses** |
| --- | --- |
| The most important concern is internal consistency. The hypothesis numbering in the hypothesis development section does not match the numbering in Table IV. This should be corrected throughout the manuscript. | Corrected. In the hypothesis-testing summary paragraph, the construct incorrectly labeled as "FC and RU (H1)" has been corrected to "PU and RU (H1)" to match Table IV, where H1 is Perceived Usefulness (PU) to Resistance to Use (RU). The numbering for the other hypotheses in this sentence (H5, H6, H8) already matched Table IV and has been left unchanged. |
| In addition, the authors should ensure that the discussion and conclusion only make claims that are directly supported by the tested model. For example, facilitating conditions and social influence were tested as predictors of resistance to use, not as direct predictors of behavioural intention. Therefore, statements claiming that these constructs have direct effects on adoption or behavioural intention should be revised. | We have revised the Abstract, Discussion, and Conclusion so that claims match what was actually tested in the model. Facilitating conditions (FC), social influence (SI), perceived ease of use (PEOU), perceived risk (PR), and technology anxiety (TA) are now described as predictors of Resistance to Use (RU), not as direct predictors of Behavioural Intention (IU)/adoption. Where the model showed FC and SI were not significant predictors of RU, this is now stated explicitly, and claims that they "hinder adoption" or have "direct effects on adoption" have been removed or reworded accordingly. We also clarified that the direct path from Resistance to Use to Behavioural Intention (H8) was not significant, so downstream claims resting on that path have been softened. |
| The statistical analysis is generally appropriate, but the reporting should be improved. The AVE threshold is incorrectly set to “<0.5,” even though the reported AVE values are acceptable because they exceed 0.50. | Corrected. The AVE threshold has been changed from "(<0.5)" to "(>0.5)", which is consistent with the reported AVE values (0.690-0.850) and standard convergent-validity guidelines. |
| The authors should also remove or clarify the claim that perceived usefulness “mediates” behavioural intention unless a formal mediation test is reported. If mediation is intended, the authors should report indirect effects, confidence intervals, and the significance of the mediation paths. If mediation is not tested, the wording should be changed to “directly influences behavioural intention. | Corrected. As no formal mediation analysis (indirect effects, bootstrapped confidence intervals, significance of indirect paths) was conducted, the claim that Perceived Usefulness (PU) "mediates" Behavioural Intention has been changed to "directly influences," both in the hypothesis-development subheading and in the H4 hypothesis statement. |
| The manuscript also needs further English editing. Although the meaning is usually understandable, many sentences remain grammatically awkward or unclear. This is particularly important because the journal does not copyedit accepted PLOS ONE manuscripts. The authors should revise the abstract, introduction, hypothesis development, discussion, and conclusion for grammatical accuracy, clarity, and consistency. | The Abstract, Introduction, Hypothesis Development, Discussion, and Conclusion have been re-reviewed and revised for grammatical accuracy and clarity, including the corrections listed in response to Reviewer 2, Comment 1 above, and the model-consistency wording revisions described above. |
| Overall, the study is potentially publishable after minor revision. The data and analysis generally support the main findings, but the manuscript requires correction of statistical wording, hypothesis numbering, interpretation of model paths, and English language presentation before final acceptance. | We thank the reviewer for this positive overall assessment and have made all requested corrections described above. |
