# Supplementary material for: Understanding the influencing factors of telemedicine service adoption in post-pandemic Bangladesh: An extended TAM and UTAUT2 model analysis
Source: PLoS One. 2026 Oct 28;21(10):e0361691. doi: 10.1371/journal.pone.0361691 (PMC13716190; doi:10.1371/journal.pone.0361691)
Supplement: Peer review history

# Peer review: Decision Letter 0

**DOI:** 10.1371/journal.pone.0361691.r001

**Authors:** Mohsin Shahzad

17 Mar 2025

Dear Dr. Makhdum,

Thank you for submitting your manuscript to PLOS ONE. After careful consideration, we feel that it has merit but does not fully meet PLOS ONE’s publication criteria as it currently stands. Therefore, we invite you to submit a revised version of the manuscript that addresses the points raised during the review process.

Please submit your revised manuscript by May 01 2025 11:59PM. If you will need more time than this to complete your revisions, please reply to this message or contact the journal office at plosone@plos.org. When you're ready to submit your revision, log on to https://www.editorialmanager.com/pone/ and select the 'Submissions Needing Revision' folder to locate your manuscript file.

- A rebuttal letter that responds to each point raised by the academic editor and reviewer(s). You should upload this letter as a separate file labeled 'Response to Reviewers'.
- A marked-up copy of your manuscript that highlights changes made to the original version. You should upload this as a separate file labeled 'Revised Manuscript with Track Changes'.
- An unmarked version of your revised paper without tracked changes. You should upload this as a separate file labeled 'Manuscript'.

If you would like to make changes to your financial disclosure, please include your updated statement in your cover letter. Guidelines for resubmitting your figure files are available below the reviewer comments at the end of this letter.

If applicable, we recommend that you deposit your laboratory protocols in protocols.io to enhance the reproducibility of your results. Protocols.io assigns your protocol its own identifier (DOI) so that it can be cited independently in the future. For instructions see: https://journals.plos.org/plosone/s/submission-guidelines#loc-laboratory-protocols. Additionally, PLOS ONE offers an option for publishing peer-reviewed Lab Protocol articles, which describe protocols hosted on protocols.io. Read more information on sharing protocols at https://plos.org/protocols?utm_medium=editorial-email&utm_source=authorletters&utm_campaign=protocols.

We look forward to receiving your revised manuscript.

Kind regards,

Mohsin Shahzad

Academic Editor

PLOS ONE

**Journal Requirements:**

1. When submitting your revision, we need you to address these additional requirements.

Please ensure that your manuscript meets PLOS ONE's style requirements, including those for file naming. The PLOS ONE style templates can be found at

https://journals.plos.org/plosone/s/file?id=wjVg/PLOSOne_formatting_sample_main_body.pdf and

https://journals.plos.org/plosone/s/file?id=ba62/PLOSOne_formatting_sample_title_authors_affiliations.pdf

2. Thank you for uploading your study's underlying data set. Unfortunately, the repository you have noted in your Data Availability statement does not qualify as an acceptable data repository according to PLOS's standards.

At this time, please upload the minimal data set necessary to replicate your study's findings to a stable, public repository (such as figshare or Dryad) and provide us with the relevant URLs, DOIs, or accession numbers that may be used to access these data. For a list of recommended repositories and additional information on PLOS standards for data deposition, please see https://journals.plos.org/plosone/s/recommended-repositories.

3. When completing the data availability statement of the submission form, you indicated that you will make your data available on acceptance. We strongly recommend all authors decide on a data sharing plan before acceptance, as the process can be lengthy and hold up publication timelines. Please note that, though access restrictions are acceptable now, your entire data will need to be made freely accessible if your manuscript is accepted for publication. This policy applies to all data except where public deposition would breach compliance with the protocol approved by your research ethics board. If you are unable to adhere to our open data policy, please kindly revise your statement to explain your reasoning and we will seek the editor's input on an exemption. Please be assured that, once you have provided your new statement, the assessment of your exemption will not hold up the peer review process.

[Note: HTML markup is below. Please do not edit.]

Reviewers' comments:

Reviewer's Responses to Questions

**Comments to the Author**

1. Is the manuscript technically sound, and do the data support the conclusions?

Reviewer #1: Yes

**********

2. Has the statistical analysis been performed appropriately and rigorously?

Reviewer #1: No

**********

3. Have the authors made all data underlying the findings in their manuscript fully available?

The PLOS Data policy

Reviewer #1: No

**********

4. Is the manuscript presented in an intelligible fashion and written in standard English?

Reviewer #1: Yes

**********

**Reviewer #1:**  Dear Author,

I have reviewed your manuscript, and the topic is highly relevant in the post-COVID era. However, several key aspects require further refinement to enhance its suitability for publication. Below are my detailed comments and recommendations:

1. Introduction: In line 89, consider incorporating examples of telemedicine adoption in different countries before presenting the rationale for focusing on Bangladesh. This will strengthen the study's contextual justification.

2. Sampling Technique: Provide details on the study area and the methodology used to determine the sample size of 276 respondents. You may refer to this article for guidance on sampling techniques, study population, and study area: PLOS ONE Article.

3. Socio-Demographic Table: The greater than (>) and less than (<) signs in the "Age of Respondents" and "Monthly Income" sections appear to be misplaced. For instance, "> 20" should likely be "< 20," and "< 50" should be "> 50." Similarly, "> 10000" should be "< 10000," and "< 50000" should be "> 50000." Please verify and correct these inconsistencies.

4. Discussion Section: Strengthen the discussion by comparing your hypothesis findings with those of similar studies to highlight key similarities or differences.

5. Referencing: Ensure all citations and references follow the APA 7 format for consistency and accuracy.

6. Sampling Justification: The explanation of the sampling technique lacks clarity. Elaborate on the rationale behind the chosen approach and justify its appropriateness for the study.

7. Structural Equation Modeling (SEM): Many tables and figures related to SEM analysis are either insufficiently analyzed or not clearly presented. Please ensure that all relevant statistical outputs are appropriately included.

8. Robustness Checks: Key methodological concerns such as endogeneity, unobserved heterogeneity, and linearity assessments appear to be overlooked. Consider conducting these robustness checks to validate your findings.

9. Theoretical Framework: The integration of TAM and UTAUT2 is not well articulated. Provide a detailed discussion on how these models complement each other and justify their combined use in the study.

10. Data Collection Approach: The use of semi-structured interviews to collect Likert scale data is unclear. Please clarify the rationale behind this methodological choice.

11. Methodology: The overall methodology section requires significant improvement in terms of clarity, rigor, and justification. Ensure that each methodological step is thoroughly explained.

12. General Structure: Refer to well-developed published papers for guidance on structuring the manuscript effectively. You may follow this paper: PLOS ONE Article.

With these revisions and improvements, I believe the paper will be in a stronger position for publication.

**********

what does this mean?). If published, this will include your full peer review and any attached files.

If you choose “no”, your identity will remain anonymous but your review may still be made public.

**Do you want your identity to be public for this peer review?**  For information about this choice, including consent withdrawal, please see our Privacy Policy

Reviewer #1: **Yes:**  Niranjan Devkota, PhD

**********

[NOTE: If reviewer comments were submitted as an attachment file, they will be attached to this email and accessible via the submission site. Please log into your account, locate the manuscript record, and check for the action link "View Attachments". If this link does not appear, there are no attachment files.]

While revising your submission, please upload your figure files to the Preflight Analysis and Conversion Engine (PACE) digital diagnostic tool, https://pacev2.apexcovantage.com/. PACE helps ensure that figures meet PLOS requirements. To use PACE, you must first register as a user. Registration is free. Then, login and navigate to the UPLOAD tab, where you will find detailed instructions on how to use the tool. If you encounter any issues or have any questions when using PACE, please email PLOS at figures@plos.org. Please note that Supporting Information files do not need this step.

# Author comment: Author response to Decision Letter 1

**DOI:** 10.1371/journal.pone.0361691.r002

1 May 2025

Dear Editor and Reviewers,

I hope you all are doing well. Thank you for your efforts in reviewing our manuscript. I hope your valuable feedback will enrich our paper. Here are the responses from the authors to your feedback.

Reviewer #1:

Dear Author,

I have reviewed your manuscript, and the topic is highly relevant in the post-COVID era. However, several key aspects require further refinement to enhance its suitability for publication. Below are my detailed comments and recommendations:

1. Introduction: In line 89, consider incorporating examples of telemedicine adoption in different countries before presenting the rationale for focusing on Bangladesh. This will strengthen the study's contextual justification.

Response:

Thank for your feedback. I think this will add value to our paper and we have incorporated some examples of telemedicine adoption in different countries before presenting the rationale for focusing on Bangladesh.

2. Sampling Technique: Provide details on the study area and the methodology used to determine the sample size of 276 respondents. You may refer to this article for guidance on sampling techniques, study population, and study area: PLOS ONE Article.

Response:

Thank for honorable reviewer. Though you haven’t received the mentioned guiding article, however we chose it as many researchers used same number of respondents to determine different factors and health issues; we have mentioned some of those papers.

3. Socio-Demographic Table: The greater than (>) and less than (<) signs in the "Age of Respondents" and "Monthly Income" sections appear to be misplaced. For instance, "> 20" should likely be "< 20," and "< 50" should be "> 50." Similarly, "> 10000" should be "< 10000," and "< 50000" should be "> 50000." Please verify and correct these inconsistencies.

Response:

Thank for the valuable feedback. We have corrected these inconsistencies.

4. Discussion Section: Strengthen the discussion by comparing your hypothesis findings with those of similar studies to highlight key similarities or differences.

Response:

Thank for the valuable feedback. We have added some comparison of my findings with similar studies.

5. Referencing: Ensure all citations and references follow the APA 7 format for consistency and accuracy.

Response:

Thank for the valuable feedback. We reviewed all the citations.

6. Sampling Justification: The explanation of the sampling technique lacks clarity. Elaborate on the rationale behind the chosen approach and justify its appropriateness for the study.

Response:

Thank for the valuable feedback. Rationale behind the chosen approach and justification has been added.

7. Structural Equation Modeling (SEM): Many tables and figures related to SEM analysis are either insufficiently analyzed or not clearly presented. Please ensure that all relevant statistical outputs are appropriately included.

Response:

Thank for your feedback. We have reviewed and tried to identify those. However it seems to us perfect and we added some articles from renowned publishers those we followed while analyzing our data. These are.

https://doi.org/10.1016/j.techsoc.2019.101212

https://doi.org/10.1186/s12911-022-01917-3

https://doi.org/10.1186/s13731 023 00336 6

8. Robustness Checks: Key methodological concerns such as endogeneity, unobserved heterogeneity, and linearity assessments appear to be overlooked. Consider conducting these robustness checks to validate your findings.

Response:

Thank you for your insightful feedback. While we recognize the importance of robustness checks, we think current methodology and model align with the research objectives and context, and we believe they adequately address the research questions. We appreciate your suggestions and will consider them for future studies.

9. Theoretical Framework: The integration of TAM and UTAUT2 is not well articulated. Provide a detailed discussion on how these models complement each other and justify their combined use in the study.

Response:

Thank you for your feedback. Discussion on how these models complement each other and justification of their combined use in the study has been added.

10. Data Collection Approach: The use of semi-structured interviews to collect Likert scale data is unclear. Please clarify the rationale behind this methodological choice.

Response:

Thank you for your insightful feedback. We apologize for mentioning interviews, it will be semi-structured survey questionnaire.

11. Methodology: The overall methodology section requires significant improvement in terms of clarity, rigor, and justification. Ensure that each methodological step is thoroughly explained.

Response:

Thank you for your insightful feedback. We have updated accordingly.

12. General Structure: Refer to well-developed published papers for guidance on structuring the manuscript effectively. You may follow this paper: PLOS ONE Article.

Response:

Thank you for your insightful feedback. Though there is no link of particular paper, however, we have followed the sample paper link provided by the Editor.

With these revisions and improvements, I believe the paper will be in a stronger position for publication.

Thank you for your kind words. We hope publishing this paper will provide insights to the policy maker of our country as well as will motivate us to conduct more research.

Attachment Submitted filename: Response to Reviewers.docx

# Peer review: Decision Letter 1

**DOI:** 10.1371/journal.pone.0361691.r003

**Authors:** Marsa Gholamzadeh

20 Aug 2025

Dear Dr. Makhdum,

Thank you for submitting your manuscript to PLOS ONE. After careful consideration, we feel that it has merit but does not fully meet PLOS ONE’s publication criteria as it currently stands. Therefore, we invite you to submit a revised version of the manuscript that addresses the points raised during the review process.

Please submit your revised manuscript by Oct 04 2025 11:59PM. If you will need more time than this to complete your revisions, please reply to this message or contact the journal office at plosone@plos.org. When you're ready to submit your revision, log on to https://www.editorialmanager.com/pone/ and select the 'Submissions Needing Revision' folder to locate your manuscript file.

- A rebuttal letter that responds to each point raised by the academic editor and reviewer(s). You should upload this letter as a separate file labeled 'Response to Reviewers'.
- A marked-up copy of your manuscript that highlights changes made to the original version. You should upload this as a separate file labeled 'Revised Manuscript with Track Changes'.
- An unmarked version of your revised paper without tracked changes. You should upload this as a separate file labeled 'Manuscript'.

If applicable, we recommend that you deposit your laboratory protocols in protocols.io to enhance the reproducibility of your results. Protocols.io assigns your protocol its own identifier (DOI) so that it can be cited independently in the future. For instructions see: https://journals.plos.org/plosone/s/submission-guidelines#loc-laboratory-protocols. Additionally, PLOS ONE offers an option for publishing peer-reviewed Lab Protocol articles, which describe protocols hosted on protocols.io. Read more information on sharing protocols at https://plos.org/protocols?utm_medium=editorial-email&utm_source=authorletters&utm_campaign=protocols.

We look forward to receiving your revised manuscript.

Kind regards,

Marsa Gholamzadeh, PhD

Academic Editor

PLOS ONE

Journal Requirements:

If the reviewer comments include a recommendation to cite specific previously published works, please review and evaluate these publications to determine whether they are relevant and should be cited. There is no requirement to cite these works unless the editor has indicated otherwise.

Please review your reference list to ensure that it is complete and correct. If you have cited papers that have been retracted, please include the rationale for doing so in the manuscript text, or remove these references and replace them with relevant current references. Any changes to the reference list should be mentioned in the rebuttal letter that accompanies your revised manuscript. If you need to cite a retracted article, indicate the article’s retracted status in the References list and also include a citation and full reference for the retraction notice.

[Note: HTML markup is below. Please do not edit.]

Reviewers' comments:

Reviewer's Responses to Questions

**Comments to the Author**

Reviewer #1: All comments have been addressed

Reviewer #2: (No Response)

**********

2. Is the manuscript technically sound, and do the data support the conclusions?

Reviewer #1: Yes

Reviewer #2: (No Response)

**********

3. Has the statistical analysis been performed appropriately and rigorously?

Reviewer #1: Yes

Reviewer #2: (No Response)

**********

4. Have the authors made all data underlying the findings in their manuscript fully available?

The PLOS Data policy

Reviewer #1: Yes

Reviewer #2: (No Response)

**********

5. Is the manuscript presented in an intelligible fashion and written in standard English?

Reviewer #1: Yes

Reviewer #2: (No Response)

**********

Reviewer #1: Dear Author,

I am glad to see that all the comments have been addressed thoroughly. However, I noticed that there is still a heading titled "Discussion and Conclusion", followed by a separate heading "Conclusion" at the end.

To maintain clarity and align with standard academic writing practices, I suggest using the heading "Discussion" only, as it conveys the intended purpose more precisely.

Best wishes,

Reviewer #2: This study presents a highly relevant investigation into the adoption of telemedicine services in Bangladesh during the post-pandemic era. This paper makes a valuable contribution to the field of digital health adoption in developing contexts, and the practical implications for healthcare providers and policymakers in Bangladesh are particularly noteworthy. I will give you some comments that will help you improve the quality of your work.

Comments:

1. There are spelling errors in some parts of the text (for example, Line 37 and Line 317). Please reread the text and correct the spelling errors.

2. Please mention keywords based on MeSH.

3. According to the submission guidelines, please abbreviate the “names of the journals” in the references.

4. Please correct the partial disorganizations in Figure 2.

5. Please include the abbreviation of each word where it is first mentioned (For example: line 93 and line 95, or line 30 and line 122).

6. You have mentioned the conclusion section separately. Therefore, it would be better to remove the word "conclusion" from the title of the discussion section (line 367).

7. Did you have any limitations in this study?

8. Please describe each table before citing it.

9. In lines 325 and 326, you wrote that around 88% of respondents have internet access to lead their daily lives in Table 2. However, this result was stated in Table 1. Please correct it.

10. Please write the number of tables and figures in the same format (Latin format (I, II, III, …) or modern format (1, 2, 3, …).

11. Please paraphrase the paragraph after Figure 2 (lines 347 to 359).

**********

what does this mean?). If published, this will include your full peer review and any attached files.

If you choose “no”, your identity will remain anonymous but your review may still be made public.

**Do you want your identity to be public for this peer review?**  For information about this choice, including consent withdrawal, please see our Privacy Policy

Reviewer #1: **Yes:**  Niranjan Devkota, PhD

Reviewer #2: No

**********

[NOTE: If reviewer comments were submitted as an attachment file, they will be attached to this email and accessible via the submission site. Please log into your account, locate the manuscript record, and check for the action link "View Attachments". If this link does not appear, there are no attachment files.]

While revising your submission, please upload your figure files to the Preflight Analysis and Conversion Engine (PACE) digital diagnostic tool, https://pacev2.apexcovantage.com/. PACE helps ensure that figures meet PLOS requirements. To use PACE, you must first register as a user. Registration is free. Then, login and navigate to the UPLOAD tab, where you will find detailed instructions on how to use the tool. If you encounter any issues or have any questions when using PACE, please email PLOS at figures@plos.org

# Author comment: Author response to Decision Letter 2

**DOI:** 10.1371/journal.pone.0361691.r004

30 Aug 2025

Author Response Letter

25 August, 2025

Dear Editor and Reviewers,

We are grateful for the reviewers’ constructive feedback. All comments have been carefully addressed, and revisions have been incorporated to improve clarity, accuracy, and presentation of the manuscript. We believe the manuscript number PONE-D-24-59687R1which entitles “Understanding the Influencing Factors of Telemedicine Service Adoption in Post-Pandemic Bangladesh: An Extended TAM and UTAUT2 Model Analysis” is now suitable for publication and hope it will be considered for acceptance.

We sincerely thank the reviewers for their constructive comments and valuable feedback. We have carefully revised the manuscript to address all the points raised. Below, we provide a detailed, point-by-point response to each reviewer’s comments.

Reviewer 1

Comment #1: All comments have been addressed

Author Response: We would like to extend our sincere appreciation to the reviewers for their valuable time, insightful comment. We appreciate the reviewer’s acknowledgment.

Comment #2: The manuscript currently has a heading titled "Discussion and Conclusion", followed by a separate heading "Conclusion". To maintain clarity, it is suggested to use the heading "Discussion" only.

Author Response: We thank the reviewer for this suggestion. We have revised the section heading to "Discussion" only, and retained a separate "Conclusion" section at the end for clarity and consistency.

Reviewer 2

Comment #1: There are spelling errors in some parts of the text (e.g., Line 37 and Line 317).

Author Response: We thank the reviewer for this suggestion. We have thoroughly proofread the manuscript and corrected the spelling errors throughout.

Comment #2: Please mention keywords based on MeSH.

Author Response: We would like to extend our sincere appreciation to the reviewers for their valuable time, insightful comment. We have revised the keywords according to the MeSH terms.

Keywords: Telemedicine, Technology Acceptance, Post-Pandemic, Attitude to Health, Cross-Sectional Studies, Bangladesh

Comment #3: According to the submission guidelines, abbreviate the names of the journals in the references.

Author Response: We are grateful for the reviewers’ constructive feedback. We have reformatted the reference list to use abbreviated journal names as per the submission guidelines.

Comment #4: Please correct the partial disorganizations in Figure 2.

Author Response: Figure 2 has been reformatted and corrected for clarity and proper alignment. Thank you so much.

Comment #5: Please include the abbreviation of each word where it is first mentioned (e.g., Line 93, Line 95, Line 30, Line 122).

Author Response: We would like to extend our sincere appreciation to the reviewers for their valuable time, insightful comment. We have ensured that all abbreviations are defined at their first occurrence in the manuscript.

Comment #6: You have mentioned the conclusion section separately. Therefore, it would be better to remove the word “conclusion” from the title of the discussion section (Line 367).

Author Response: We would like to extend our sincere appreciation to the reviewers for their valuable time, insightful comment. As suggested, we have modified the heading to 'Discussion' only. The Conclusion remains as a separate final section.

Comment #7: Did you have any limitations in this study?

Author Response: We have added a Limitations subsection in the Discussion section to acknowledge the study’s limitations. We are grateful for the reviewers’ constructive feedback.

Comment #8: Please describe each table before citing it.

Author Response: We have revised the text to provide a descriptive introduction for each table before referencing it.

Comment #9: In Lines 325–326, it is stated that around 88% of respondents have internet access in Table 2, but this result appears in Table 1. Please correct.

Author Response: We apologize for this oversight. The error has been corrected, and the description now refers to the correct table. We would like to extend our sincere appreciation to the reviewers for their valuable time, insightful comment.

Comment #10: Please write the number of tables and figures in the same format (Latin I, II, III… or modern 1, 2, 3…).

Author Response: We have standardized the numbering format for tables and figures to Latin I, II, III. We are grateful for the reviewers’ constructive feedback.

Comment #11: Please paraphrase the paragraph after Figure 2 (Lines 347–359).

Author Response: The paragraph after Figure 2 has been paraphrased and revised for better readability and originality. Thank you so much.

Attachment Submitted filename: Author_Response_Letter Telemedicine.docx

# Peer review: Decision Letter 2

**DOI:** 10.1371/journal.pone.0361691.r005

**Authors:** Andry Alamsyah

17 Jun 2026

Dear Dr. Makhdum,

Thank you for submitting your manuscript to PLOS ONE. After careful consideration, we feel that it has merit but does not fully meet PLOS ONE’s publication criteria as it currently stands. Therefore, we invite you to submit a revised version of the manuscript that addresses the points raised during the review process.

Please submit your revised manuscript by Aug 01 2026 11:59PM. If you will need more time than this to complete your revisions, please reply to this message or contact the journal office at plosone@plos.org. When you're ready to submit your revision, log on to https://www.editorialmanager.com/pone/ and select the 'Submissions Needing Revision' folder to locate your manuscript file.

- A letter that responds to each point raised by the academic editor and reviewer(s). You should upload this letter as a separate file labeled 'Response to Reviewers'.
- A marked-up copy of your manuscript that highlights changes made to the original version. You should upload this as a separate file labeled 'Revised Manuscript with Track Changes'.
- An unmarked version of your revised paper without tracked changes. You should upload this as a separate file labeled 'Manuscript'.

If you would like to make changes to your financial disclosure, please include your updated statement in your cover letter. Guidelines for resubmitting your figure files are available below the reviewer comments at the end of this letter.

If applicable, we recommend that you deposit your laboratory protocols in protocols.io to enhance the reproducibility of your results. Protocols.io assigns your protocol its own identifier (DOI) so that it can be cited independently in the future. For instructions see: https://journals.plos.org/plosone/s/submission-guidelines#loc-laboratory-protocols. Additionally, PLOS ONE offers an option for publishing peer-reviewed Lab Protocol articles, which describe protocols hosted on protocols.io. Read more information on sharing protocols at https://plos.org/protocols?utm_medium=editorial-email&utm_source=authorletters&utm_campaign=protocols.

As the corresponding author, your ORCID iD is verified in the submission system and will appear in the published article. PLOS supports the use of ORCID, and we encourage all coauthors to register for an ORCID iD and use it as well. Please encourage your coauthors to verify their ORCID iD within the submission system before final acceptance, as unverified ORCID iDs will not appear in the published article. *Only*  the individual author can complete the verification step; PLOS staff *cannot*  verify ORCID iDs on behalf of authors.

We look forward to receiving your revised manuscript.

Kind regards,

Andry Alamsyah

Academic Editor

PLOS One

Journal Requirements:

If the reviewer comments include a recommendation to cite specific previously published works, please review and evaluate these publications to determine whether they are relevant and should be cited. There is no requirement to cite these works unless the editor has indicated otherwise.

Additional Editor Comments:

Reviewers still have some concerns about your revision, and some were not addressed (particularly those from Reviewer 2). Reviewer 3 has given you Minor Revisions.

Please address the reviewers' concerns thoroughly.

Best regards

[Note: HTML markup is below. Please do not edit.]

Reviewers' comments:

Reviewer's Responses to Questions

**Comments to the Author**

Reviewer #1: All comments have been addressed

Reviewer #2: (No Response)

Reviewer #3: (No Response)

**********

2. Is the manuscript technically sound, and do the data support the conclusions?

Reviewer #1: Yes

Reviewer #2: (No Response)

Reviewer #3: Partly

**********

3. Has the statistical analysis been performed appropriately and rigorously?

Reviewer #1: Yes

Reviewer #2: (No Response)

Reviewer #3: Yes

**********

4. Have the authors made all data underlying the findings in their manuscript fully available?

The PLOS Data policy

Reviewer #1: Yes

Reviewer #2: (No Response)

Reviewer #3: Yes

**********

5. Is the manuscript presented in an intelligible fashion and written in standard English?

Reviewer #1: Yes

Reviewer #2: (No Response)

Reviewer #3: Yes

**********

Reviewer #1: Dear Author,

I am glad to see that all the comments have been addressed thoroughly.

Now, I have no further issues in this manuscript.

Best wishes,

Reviewer #2: Some comments were not addressed: Comments 1, 2, 3, 5, 7, 8, and 10! These comments are not suggestions and are provided to improve your paper. Please investigate them carefully again.

Reviewer #3: The manuscript examines an important and timely issue: factors influencing telemedicine service adoption in post-pandemic Bangladesh. The topic is relevant to digital health, technology acceptance, and healthcare accessibility, especially in rural and underserved contexts. The use of an extended TAM and UTAUT2 framework is appropriate, and the study provides useful empirical evidence regarding the role of trust, perceived usefulness, perceived ease of use, perceived risk, technology anxiety, and resistance to use.

Several issues still need correction before it can be accepted. The most important concern is internal consistency. The hypothesis numbering in the hypothesis development section does not match the numbering in Table IV. This should be corrected throughout the manuscript. In addition, the authors should ensure that the discussion and conclusion only make claims that are directly supported by the tested model. For example, facilitating conditions and social influence were tested as predictors of resistance to use, not as direct predictors of behavioural intention. Therefore, statements claiming that these constructs have direct effects on adoption or behavioural intention should be revised.

The statistical analysis is generally appropriate, but the reporting should be improved. The AVE threshold is incorrectly set to “<0.5,” even though the reported AVE values are acceptable because they exceed 0.50. The authors should also remove or clarify the claim that perceived usefulness “mediates” behavioural intention unless a formal mediation test is reported. If mediation is intended, the authors should report indirect effects, confidence intervals, and the significance of the mediation paths. If mediation is not tested, the wording should be changed to “directly influences behavioural intention.”

The manuscript also needs further English editing. Although the meaning is usually understandable, many sentences remain grammatically awkward or unclear. This is particularly important because the journal does not copyedit accepted PLOS ONE manuscripts. The authors should revise the abstract, introduction, hypothesis development, discussion, and conclusion for grammatical accuracy, clarity, and consistency.

Overall, the study is potentially publishable after minor revision. The data and analysis generally support the main findings, but the manuscript requires correction of statistical wording, hypothesis numbering, interpretation of model paths, and English language presentation before final acceptance.

**********

what does this mean?). If published, this will include your full peer review and any attached files.

If you choose “no”, your identity will remain anonymous but your review may still be made public.

**Do you want your identity to be public for this peer review?**  For information about this choice, including consent withdrawal, please see our Privacy Policy

Reviewer #1: **Yes:**  Niranjan Devkota, PhD

Reviewer #2: No

Reviewer #3: No

**********

[NOTE: If reviewer comments were submitted as an attachment file, they will be attached to this email and accessible via the submission site. Please log into your account, locate the manuscript record, and check for the action link "View Attachments". If this link does not appear, there are no attachment files.]

To ensure your figures meet our technical requirements, please review our figure guidelines: https://journals.plos.org/plosone/s/figures

You may also use PLOS’s free figure tool, NAAS, to help you prepare publication quality figures: https://journals.plos.org/plosone/s/figures#loc-tools-for-figure-preparation.

NAAS will assess whether your figures meet our technical requirements by comparing each figure against our figure specifications.

# Author comment: Author response to Decision Letter 3

**DOI:** 10.1371/journal.pone.0361691.r006

17 Jul 2026

Response to Reviewers

Reviewer 2

Comments Authors’ Responses

Some comments were not addressed: Comments 1, 2, 3, 5, 7, 8, and 10! These comments are not suggestions and are provided to improve your paper. Please investigate them carefully again. We thank the reviewer for flagging these. We have re-reviewed the manuscript carefully and confirmed/completed the following revisions (all marked as tracked changes in the resubmitted file).

Comment 1 There are spelling errors in some parts of the text (for example, Line 37 and Line 317). Please reread the text and correct the spelling errors.

- We have proofread the manuscript again and corrected the spelling and grammatical errors identified, including in the Abstract and Discussion sections. Additional minor corrections (e.g., "finding highpoints" to "findings highlight", "research's" to "research has been", "believes" to "beliefs", "furthermost established" to "most well-established", duplicated "mobile mobile", "For examples" to "For example", subject-verb agreement in "Telemedical services covers") have also been corrected throughout the manuscript. All changes are tracked.

Comment 2 Please mention keywords based on MeSH.

- The keywords have been revised to MeSH-based terms: Telemedicine; Patient Acceptance of Health Care; Health Services Accessibility; Attitude to Health; Cross-Sectional Studies; Bangladesh.

Comment 3 According to the submission guidelines, please abbreviate the “names of the journals” in the references.

- All references have been reviewed and journal names abbreviated according to standard NLM/PubMed style, consistent with PLOS ONE submission guidelines (e.g., "Technological Forecasting and Social Change" to "Technol Forecast Soc Change"; "MIS Quarterly" to "MIS Q"; "Journal of Business Research" to "J Bus Res"; "Journal of Retailing and Consumer Services" to "J Retail Consum Serv"; "Technology in Society" to "Technol Soc"; "International Journal of Telemedicine and Applications" to "Int J Telemed Appl"; "International Journal of Medical Informatics" to "Int J Med Inform").

Comment 5 Please include the abbreviation of each word where it is first mentioned (For example: line 93 and line 95, or line 30 and line 122).

- We have re-audited every abbreviation in the manuscript. Two abbreviations used without a prior full-form definition have now been corrected: "AVE" is now introduced as "Average Variance Extracted (AVE)" and "HTMT" as "Heterotrait-Monotrait ratio (HTMT)" at their first mention in the Results section (previously HTMT was only spelled out in the Table III caption, which appeared after its first use in the text). All other abbreviations (TAM, UTAUT2, PU, PEOU, TA, RU, FC, SI, PR, IU, TLS, PLS-SEM, mHealth) were checked and confirmed to be defined in full at their first occurrence in the text.

Comment 7 Did you have any limitations in this study?

- Yes. A "Limitations and Future Directions" section is included before the Conclusion, noting the cross-sectional design, convenience/snowball sampling, and self-report bias as limitations, with corresponding suggestions for future research.

Comment 8 Please describe each table before citing it.

- A brief lead-in sentence introducing and describing the content of each table (Table I-IV) has been added immediately before the table appears, so that each table is described before it is cited/presented.

Comment 10 Please write the number of tables and figures in the same format (Latin format (I, II, III, ..) or modern format (1, 2, 3, ..).

- Figure numbering has been changed from Arabic (1, 2) to Roman numerals (I, II) so that it is consistent with the Table numbering format (Table I-IV), as requested.

Reviewer 3

The most important concern is internal consistency. The hypothesis numbering in the hypothesis development section does not match the numbering in Table IV. This should be corrected throughout the manuscript.

- Corrected. In the hypothesis-testing summary paragraph, the construct incorrectly labeled as "FC and RU (H1)" has been corrected to "PU and RU (H1)" to match Table IV, where H1 is Perceived Usefulness (PU) to Resistance to Use (RU). The numbering for the other hypotheses in this sentence (H5, H6, H8) already matched Table IV and has been left unchanged.

In addition, the authors should ensure that the discussion and conclusion only make claims that are directly supported by the tested model. For example, facilitating conditions and social influence were tested as predictors of resistance to use, not as direct predictors of behavioural intention. Therefore, statements claiming that these constructs have direct effects on adoption or behavioural intention should be revised.

- We have revised the Abstract, Discussion, and Conclusion so that claims match what was actually tested in the model. Facilitating conditions (FC), social influence (SI), perceived ease of use (PEOU), perceived risk (PR), and technology anxiety (TA) are now described as predictors of Resistance to Use (RU), not as direct predictors of Behavioural Intention (IU)/adoption. Where the model showed FC and SI were not significant predictors of RU, this is now stated explicitly, and claims that they "hinder adoption" or have "direct effects on adoption" have been removed or reworded accordingly. We also clarified that the direct path from Resistance to Use to Behavioural Intention (H8) was not significant, so downstream claims resting on that path have been softened.

The statistical analysis is generally appropriate, but the reporting should be improved. The AVE threshold is incorrectly set to “<0.5,” even though the reported AVE values are acceptable because they exceed 0.50.

- Corrected. The AVE threshold has been changed from "(<0.5)" to "(>0.5)", which is consistent with the reported AVE values (0.690-0.850) and standard convergent-validity guidelines.

The authors should also remove or clarify the claim that perceived usefulness “mediates” behavioural intention unless a formal mediation test is reported. If mediation is intended, the authors should report indirect effects, confidence intervals, and the significance of the mediation paths. If mediation is not tested, the wording should be changed to “directly influences behavioural intention.

- Corrected. As no formal mediation analysis (indirect effects, bootstrapped confidence intervals, significance of indirect paths) was conducted, the claim that Perceived Usefulness (PU) "mediates" Behavioural Intention has been changed to "directly influences," both in the hypothesis-development subheading and in the H4 hypothesis statement.

The manuscript also needs further English editing. Although the meaning is usually understandable, many sentences remain grammatically awkward or unclear. This is particularly important because the journal does not copyedit accepted PLOS ONE manuscripts. The authors should revise the abstract, introduction, hypothesis development, discussion, and conclusion for grammatical accuracy, clarity, and consistency.

- The Abstract, Introduction, Hypothesis Development, Discussion, and Conclusion have been re-reviewed and revised for grammatical accuracy and clarity, including the corrections listed in response to Reviewer 2, Comment 1 above, and the model-consistency wording revisions described above.

Overall, the study is potentially publishable after minor revision. The data and analysis generally support the main findings, but the manuscript requires correction of statistical wording, hypothesis numbering, interpretation of model paths, and English language presentation before final acceptance.

- We thank the reviewer for this positive overall assessment and have made all requested corrections described above.

Attachment Submitted filename: Response_to_Reviewers_auresp_3.docx

# Peer review: Decision Letter 3

**DOI:** 10.1371/journal.pone.0361691.r007

**Authors:** Syed Hamid Hussain Madni

14 Oct 2026

Understanding the Influencing Factors of Telemedicine Service Adoption in Post-Pandemic Bangladesh: An Extended TAM and UTAUT2 Model Analysis

PONE-D-24-59687R3

Dear Dr. Makhdum,

We’re pleased to inform you that your manuscript has been judged scientifically suitable for publication and will be formally accepted for publication once it meets all outstanding technical requirements.

Within one week, you’ll receive an e-mail detailing the required amendments. When these have been addressed, you’ll receive a formal acceptance letter and your manuscript will be scheduled for publication.

An invoice will be generated when your article is formally accepted. Please note, if your institution has a publishing partnership with PLOS and your article meets the relevant criteria, all or part of your publication costs will be covered. Please make sure your user information is up-to-date by logging into Editorial Manager at Editorial Manager® and clicking the ‘Update My Information' link at the top of the page. For questions related to billing, please contact billing support.

If your institution or institutions have a press office, please notify them about your upcoming paper to help maximize its impact. If they’ll be preparing press materials, please inform our press team as soon as possible -- no later than 48 hours after receiving the formal acceptance. Your manuscript will remain under strict press embargo until 2 pm Eastern Time on the date of publication. For more information, please contact onepress@plos.org.

Kind regards,

Syed Hamid Hussain Madni

Academic Editor

PLOS One

Additional Editor Comments (optional):

Reviewers' comments:

Reviewer's Responses to Questions

**Comments to the Author**

Reviewer #3: All comments have been addressed

Reviewer #4: All comments have been addressed

**********

2. Is the manuscript technically sound, and do the data support the conclusions?

Reviewer #3: Yes

Reviewer #4: Yes

**********

3. Has the statistical analysis been performed appropriately and rigorously?

Reviewer #3: Yes

Reviewer #4: Yes

**********

4. Have the authors made all data underlying the findings in their manuscript fully available?

The PLOS Data policy

Reviewer #3: Yes

Reviewer #4: Yes

**********

5. Is the manuscript presented in an intelligible fashion and written in standard English?

Reviewer #3: Yes

Reviewer #4: Yes

**********

Reviewer #3: 1. The authors have adequately addressed the comments and suggestions raised in the previous round of review. The revisions have improved the clarity, consistency, and overall quality of the manuscript.

2. The manuscript is technically sound, and the data presented adequately support the main findings and conclusions. The interpretation of the results is generally consistent with the evidence provided in the study.

3. The statistical analyses have been performed appropriately and with sufficient rigor. The analytical procedures are suitable for the research objectives, and the results are reported and interpreted appropriately.

4. The authors have provided sufficient information regarding the data underlying the findings presented in the manuscript. The level of data availability is adequate to support the transparency and reproducibility of the reported results.

5. The manuscript is presented in an intelligible and well-structured manner. The writing is generally clear and uses standard academic English, with no major language issues that would hinder readers’ understanding of the manuscript.

Reviewer #4: Cross check the references, some references are either not cited, or not available in bibliography

Add paper organization is at the end of Introduction section.

**********

what does this mean?). If published, this will include your full peer review and any attached files.

If you choose “no”, your identity will remain anonymous but your review may still be made public.

**Do you want your identity to be public for this peer review?**  For information about this choice, including consent withdrawal, please see our Privacy Policy

Reviewer #3: No

Reviewer #4: No

**********

# Editor report: Acceptance letter

**DOI:** 10.1371/journal.pone.0361691.r008

**Authors:** Syed Hamid Hussain Madni

PONE-D-24-59687R3

PLOS One

Dear Dr. Makhdum,

I'm pleased to inform you that your manuscript has been deemed suitable for publication in PLOS One. Congratulations! Your manuscript is now being handed over to our production team.

At this stage, our production department will prepare your paper for publication. This includes ensuring the following:

* All references, tables, and figures are properly cited

* All relevant supporting information is included in the manuscript submission,

* There are no issues that prevent the paper from being properly typeset

You will receive further instructions from the production team, including instructions on how to review your proof when it is ready. Please keep in mind that we are working through a large volume of accepted articles, so please give us a few days to review your paper and let you know the next and final steps.

Lastly, if your institution or institutions have a press office, please let them know about your upcoming paper now to help maximize its impact. If they'll be preparing press materials, please inform our press team within the next 48 hours. Your manuscript will remain under strict press embargo until 2 pm Eastern Time on the date of publication. For more information, please contact onepress@plos.org.

You will receive an invoice from PLOS for your publication fee after your manuscript has reached the completed accept phase. If you receive an email requesting payment before acceptance or for any other service, this may be a phishing scheme. Learn how to identify phishing emails and protect your accounts at https://explore.plos.org/phishing.

If we can help with anything else, please email us at customercare@plos.org.

Thank you for submitting your work to PLOS One and supporting open access.

Kind regards,

PLOS One Editorial Office Staff

on behalf of

Dr. Syed Hamid Hussain Madni

Academic Editor

PLOS One
